# Supplementary figures and images for: Detailed comparison of two popular variant calling packages for exome and targeted exon studies
Source: PeerJ. 2014 Sep 30;2:e600. doi: 10.7717/peerj.600 (PMC4184249; doi:10.7717/peerj.600)

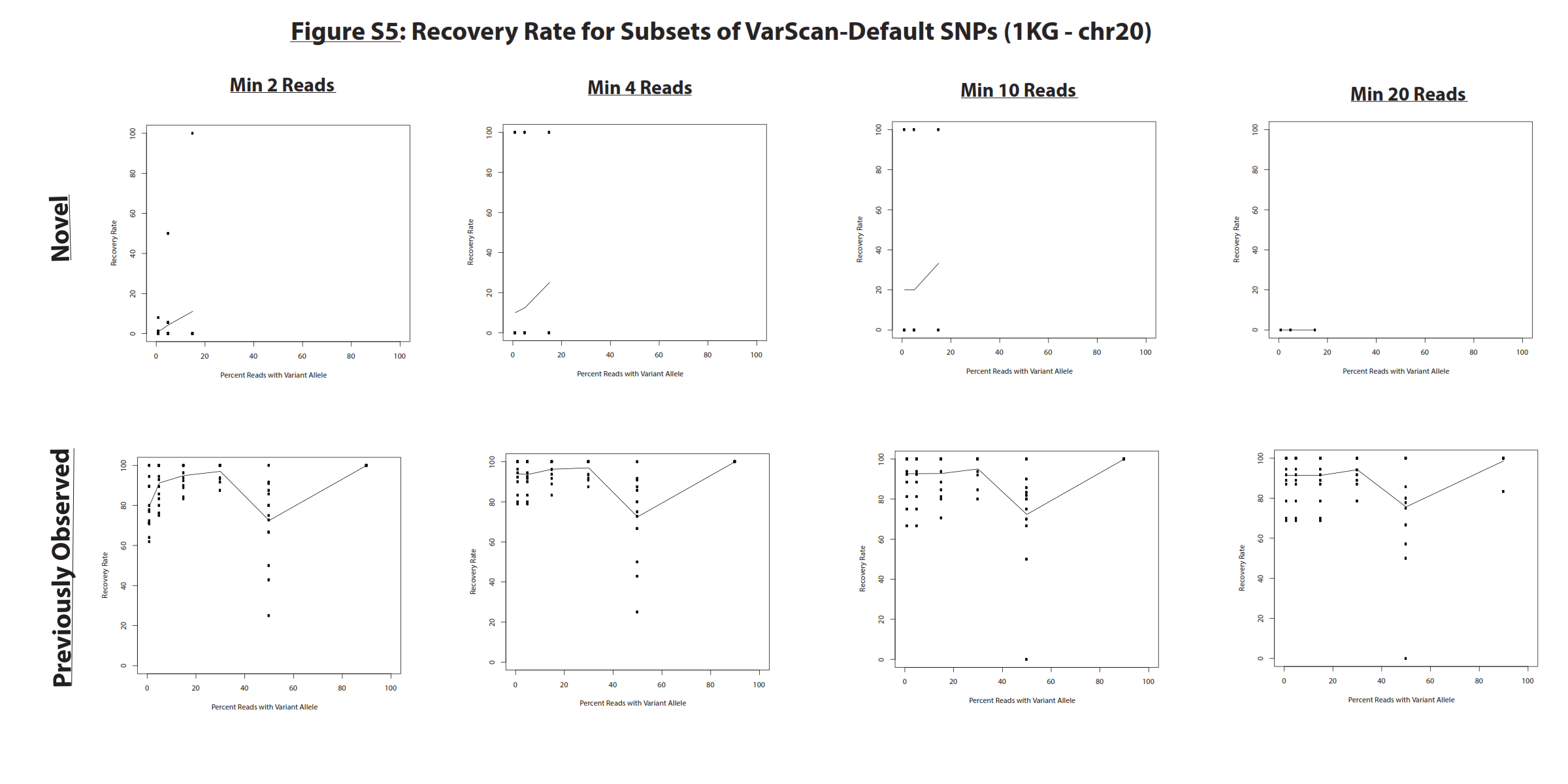

Supplement: Figure S5 — Percent recovery of targeted exon SNPs in the paired exome sample is shown on the y-axis. Recovery rates are calculated separately for novel versus previously observed variants, and the minimum number of reads containing the variant sequence is also varied (between 2, 4, 10, and 20 reads). For each type of variant and minimum number of reads containing a variant, the concordance rate is compared to the proportion of reads containing the variant that was called with VarScan using default parameters (with a minimum of 1%, 5%, 15%, 30%, 50%, and 90% supporting reads). Each dot shows the recovery for an individual sample, overlaid with line representing the average recovery rate at each threshold. Most samples did not contain novel variants present in at least 20 reads (Table S11), within targeted regions of chromosome 20. Notice that the recovery rate is consistently better for previously observed variants and that it usually improves with larger percentages of supporting reads (except for exactly 50%, which splits up the heterozygous peak and the decrease in concordance is probably due to variants that marginally do or do not meet this criteria). [file peerj-02-600-s005.png]
