# Supplementary material for: Detailed comparison of two popular variant calling packages for exome and targeted exon studies
Source: PeerJ. 2014 Sep 30;2:e600. doi: 10.7717/peerj.600 (PMC4184249; doi:10.7717/peerj.600)
Supplement: Table S1 — BI = Broad Institute, SC = Sanger Institute [file peerj-02-600-s020.doc]

**Table S1: Alignment Statistics for Targeted Exon Datasets**

| **Sample** | **SubjectID** | **Population Code** | **Sequencing Center** | **Total.Reads** | **Unique.Reads** | **Aligned.Unique.Reads** | **Percent.Duplicates** | **Num.Target.Reads** | **Percent.Target.Reads** | **Fold.Coverage** |
| --- | --- | --- | --- | --- | --- | --- | --- | --- | --- | --- |
| ERR002985 | NA11893 | CEU | SC | 6752808 | 6251978 | 6251978 | 4.6341 | 2399471 | 0.383794 | 28.31594 |
| ERR002993 | NA12287 | CEU | SC | 8053498 | 7156175 | 7156175 | 9.5632 | 3913511 | 0.546872 | 48.22572 |
| ERR002994 | NA18532 | CHB | SC | 5755536 | 5327366 | 5327366 | 5.4687 | 2342235 | 0.439661 | 28.3966 |
| ERR003014 | NA18566 | CHB | SC | 22673582 | 15956505 | 15956505 | 25.4061 | 8659691 | 0.542706 | 106.1586 |
| ERR003016 | NA18505 | YRI | SC | 15264922 | 10921034 | 10921034 | 21.5632 | 3112549 | 0.285005 | 30.73711 |
| ERR004078 | NA18858 | YRI | SC | 36157144 | 17473260 | 17473260 | 50.4928 | 9588713 | 0.548765 | 117.2291 |
| ERR004084 | NA19087 | JPT | SC | 21960498 | 16620631 | 16620631 | 18.5298 | 9348955 | 0.562491 | 105.1525 |
| SRR013632 | NA19000 | JPT | BI | 37927466 | 5567881 | 5567881 | 45.6943 | 1967728 | 0.353407 | 62.65048 |
| SRR013635 | NA19058 | JPT | BI | 29035684 | 4506922 | 4506922 | 71.6265 | 1592615 | 0.353371 | 54.03871 |
| SRR013654 | NA18637 | CHB | BI | 20862902 | 6446002 | 6446002 | 55.9668 | 3081962 | 0.47812 | 99.38469 |
| SRR013709 | NA18637 | CHB | BI | 18277230 | 6787208 | 6787208 | 55.4755 | 3160422 | 0.465644 | 109.5487 |
| SRR017908 | NA18510 | YRI | BI | 33564496 | 21680063 | 21680063 | 23.1265 | 6376149 | 0.294102 | 90.47467 |
| SRR017914 | NA18870 | YRI | BI | 19412854 | 1962411 | 1962411 | 75.718 | 1062981 | 0.541671 | 32.82901 |
| SRR018122 | NA18510 | YRI | BI | 44323302 | 10766392 | 10766392 | 54.121 | 3088974 | 0.286909 | 91.09718 |

BI=Broad Institute, SC=Sanger Institute
