# Supplementary material for: Detailed comparison of two popular variant calling packages for exome and targeted exon studies
Source: PeerJ. 2014 Sep 30;2:e600. doi: 10.7717/peerj.600 (PMC4184249; doi:10.7717/peerj.600)
Supplement: Table S2 — BGI = Bejing Genome Institute, BI = Broad Institute, BCM = Baylor College of Medicine [file peerj-02-600-s021.doc]

**Table S2: Alignment Statistics for 1KG Exome Samples**

| **Sample** | **SubjectID** | **Population Code** | **Sequencing Center** | **Total.Reads** | **Unique.Reads** | **Aligned.Unique.Reads** | **Percent.Duplicates** | **Num.Target.Reads** | **Percent.Target.Reads** | **Fold.Coverage** |
| --- | --- | --- | --- | --- | --- | --- | --- | --- | --- | --- |
| ERR031862 | NA18566 | CHB | BGI | 107236256 | 99964352 | 99964352 | 3.875 | 77145589 | 0.771731 | 84.690014 |
| ERR031931 | NA18637 | CHB | BGI | 87861320 | 80912035 | 80912035 | 3.9217 | 60695596 | 0.750143 | 68.446477 |
| ERR031956 | NA18532 | CHB | BGI | 163139168 | 142736163 | 142736163 | 4.9965 | 90964614 | 0.637292 | 99.816998 |
| ERR034519 | NA11893 | CEU | BGI | 123904406 | 115104093 | 115104093 | 5.8383 | 96862971 | 0.841525 | 106.665867 |
| ERR034546 | NA12287 | CEU | BGI | 121397670 | 112760024 | 112760024 | 5.7183 | 95051400 | 0.842953 | 106.310326 |
| ERR034553 | NA18858 | YRI | BGI | 136831266 | 127388622 | 127388622 | 5.6947 | 104586440 | 0.821003 | 116.267036 |
| ERR034606 | NA19087 | JPT | BGI | 138667910 | 129439282 | 129439282 | 5.0453 | 112167033 | 0.866561 | 128.647296 |
| SRR098520 | NA18510 | YRI | BI | 246717590 | 195440467 | 195440467 | 9.7828 | 95358921 | 0.487918 | 103.156061 |
| SRR099528 | NA19000 | JPT | BI | 235978060 | 156444797 | 156444797 | 21.0198 | 145164814 | 0.927898 | 168.938437 |
| SRR099541 | NA19058 | JPT | BI | 193628096 | 141751074 | 141751074 | 19.1025 | 131655562 | 0.92878 | 158.288986 |
| SRR100031 | NA18870 | YRI | BI | 185401654 | 137321758 | 137321758 | 16.1923 | 123814652 | 0.901639 | 146.019959 |
| SRX237141 | NA18505 | YRI | BCM | 96134084 | 87067067 | 87067067 | 6.6593 | 71704779 | 0.823558 | 110.916259 |

BGI=Bejing Genome Institute, BI=Broad Institute, BCM=Baylor College of Medicine
