# Supplementary material for: Detailed comparison of two popular variant calling packages for exome and targeted exon studies
Source: PeerJ. 2014 Sep 30;2:e600. doi: 10.7717/peerj.600 (PMC4184249; doi:10.7717/peerj.600)
Supplement: Table S3 [file peerj-02-600-s022.doc]

**Table S3: Alignment Statistics for SRP019719 Illumina Exome Samples**

| **Sample** | **Total.Reads** | **Unique.Reads** | **Aligned.Unique.Reads** | **Percent.Duplicates** | **Num.Target.Reads** | **Percent.Target.Reads** | **Fold.Coverage** |
| --- | --- | --- | --- | --- | --- | --- | --- |
| SRR796868 | 75290846 | 63968610 | 63968610 | 13.4303 | 54690858 | 0.854964 | 67.877497 |
| SRR796869 | 87228590 | 74716290 | 74716290 | 12.8024 | 64703858 | 0.865994 | 81.235152 |
| SRR796870 | 106757982 | 55348476 | 55348476 | 46.5354 | 46722028 | 0.844143 | 59.933053 |
| SRR796871 | 138779950 | 114673108 | 114673108 | 10.3588 | 75022472 | 0.654229 | 108.788471 |
| SRR796872 | 71205444 | 58400729 | 58400729 | 15.8355 | 50415363 | 0.863266 | 63.389211 |
| SRR796873 | 161898170 | 132353155 | 132353155 | 11.5642 | 86853713 | 0.656227 | 126.97665 |
| SRR796874 | 156985870 | 134700194 | 134700194 | 8.3527 | 85436830 | 0.634274 | 122.770284 |
| SRR796875 | 83850140 | 70718402 | 70718402 | 13.8891 | 61215333 | 0.865621 | 76.927985 |
| SRR796876 | 86066920 | 68365933 | 68365933 | 16.839 | 58372269 | 0.853821 | 75.54341 |
| SRR796877 | 80048272 | 67668538 | 67668538 | 12.2677 | 57873381 | 0.855248 | 72.914859 |
| SRR796878 | 84870050 | 71100854 | 71100854 | 13.9621 | 60539746 | 0.851463 | 77.09932 |
| SRR796879 | 136449194 | 90205236 | 90205236 | 31.181 | 75469489 | 0.836642 | 93.322 |
| SRR796880 | 94243560 | 76272479 | 76272479 | 15.8197 | 65533924 | 0.859208 | 83.715905 |
| SRR796881 | 79964246 | 58195062 | 58195062 | 15.4234 | 51054586 | 0.877301 | 77.17152 |
| SRX265476 | 80381332 | 77102445 | 77102445 | 0.4835 | 65856051 | 0.854137 | 84.063762 |
