# Supplementary material for: Detailed comparison of two popular variant calling packages for exome and targeted exon studies
Source: PeerJ. 2014 Sep 30;2:e600. doi: 10.7717/peerj.600 (PMC4184249; doi:10.7717/peerj.600)
Supplement: Table S4 [file peerj-02-600-s023.doc]

**Table S4: Recovery of Targeted Exon SNPs in Exome Data for NA18510 (1KG - chr20)**

| **Variant.Caller** | **Preprocessing** | **SRR017908** | **SRR018122** |
| --- | --- | --- | --- |
| VarScan.custom | Indel.Realignment | 100 | 100 |
| VarScan.custom | None | 100 | 100 |
| GATK.Unified.HQ | Base.Recalibration | 100 | 88 |
| GATK.Unified.HQ | Full.Pipeline | 100 | 88 |
| GATK.Haplotype.all | Base.Recalibration | 100 | 84.21052632 |
| GATK.Haplotype.all | Full.Pipeline | 100 | 84.21052632 |
| GATK.Haplotype.all | Indel.Realignment | 100 | 83.33333333 |
| GATK.Haplotype.all | None | 100 | 83.33333333 |
| GATK.Haplotype.HQ | Base.Recalibration | 100 | 83.33333333 |
| GATK.Haplotype.HQ | Full.Pipeline | 100 | 83.33333333 |
| GATK.Haplotype.HQ | Indel.Realignment | 100 | 83.33333333 |
| GATK.Haplotype.HQ | None | 100 | 83.33333333 |
| VarScan.custom | Base.Recalibration | 100 | 83.33333333 |
| VarScan.custom | Full.Pipeline | 100 | 83.33333333 |
| GATK.Unified.all | None | 96.55172414 | 81.25 |
| GATK.Unified.HQ | Indel.Realignment | 96.55172414 | 81.25 |
| GATK.Unified.HQ | None | 96.55172414 | 81.25 |
| GATK.Unified.all | Base.Recalibration | 96.42857143 | 88 |
| GATK.Unified.all | Full.Pipeline | 96.42857143 | 88 |
| VarScan.pvalue | Indel.Realignment | 96 | 80 |
| VarScan.pvalue | None | 96 | 80 |
| GATK.Unified.all | Indel.Realignment | 94.11764706 | 75 |
| VarScan.pvalue | Base.Recalibration | 92.59259259 | 83.33333333 |
| VarScan.pvalue | Full.Pipeline | 92.59259259 | 83.33333333 |
| VarScan | Indel.Realignment | 10.63829787 | 22.80701754 |
| VarScan | None | 10.63829787 | 22.80701754 |
| VarScan | Full.Pipeline | 7.004830918 | 7.258064516 |
| VarScan | Base.Recalibration | 7.004830918 | 7.228915663 |
