# Supplementary material for: Detailed comparison of two popular variant calling packages for exome and targeted exon studies
Source: PeerJ. 2014 Sep 30;2:e600. doi: 10.7717/peerj.600 (PMC4184249; doi:10.7717/peerj.600)
Supplement: Table S5 [file peerj-02-600-s024.doc]

**Table S5**: Recovery of Targeted Exon SNPs in Exome Data for NA18637 (1KG - chr20)

| **Variant.Caller** | **Preprocessing** | **SRR013654** | **SRR013709** |
| --- | --- | --- | --- |
| GATK.Haplotype.all | Base.Recalibration | 100 | 100 |
| GATK.Haplotype.all | Full.Pipeline | 100 | 100 |
| GATK.Haplotype.HQ | Base.Recalibration | 100 | 100 |
| GATK.Haplotype.HQ | Full.Pipeline | 100 | 100 |
| GATK.Haplotype.HQ | Indel.Realignment | 100 | 94.73684211 |
| GATK.Haplotype.HQ | None | 100 | 94.73684211 |
| GATK.Haplotype.all | Indel.Realignment | 94.11764706 | 90 |
| GATK.Haplotype.all | None | 94.11764706 | 90 |
| VarScan.custom | Base.Recalibration | 93.33333333 | 93.33333333 |
| VarScan.custom | Full.Pipeline | 93.33333333 | 93.33333333 |
| VarScan.custom | Indel.Realignment | 93.33333333 | 93.33333333 |
| VarScan.custom | None | 93.33333333 | 93.33333333 |
| GATK.Unified.HQ | Base.Recalibration | 88.88888889 | 80.95238095 |
| GATK.Unified.HQ | Full.Pipeline | 88.88888889 | 80.95238095 |
| GATK.Unified.all | Base.Recalibration | 85.71428571 | 81.81818182 |
| GATK.Unified.all | Full.Pipeline | 85.71428571 | 81.81818182 |
| GATK.Unified.all | None | 85 | 72 |
| GATK.Unified.HQ | Indel.Realignment | 85 | 72 |
| GATK.Unified.HQ | None | 85 | 72 |
| GATK.Unified.all | Indel.Realignment | 72 | 69.23076923 |
| VarScan.pvalue | Base.Recalibration | 42.85714286 | 53.33333333 |
| VarScan.pvalue | Full.Pipeline | 42.85714286 | 53.33333333 |
| VarScan.pvalue | Indel.Realignment | 10.48951049 | 45.45454545 |
| VarScan.pvalue | None | 10.48951049 | 45.45454545 |
| VarScan | Base.Recalibration | 5.647840532 | 5.806451613 |
| VarScan | Full.Pipeline | 5.647840532 | 5.806451613 |
| VarScan | Indel.Realignment | 1.189127973 | 6.463878327 |
| VarScan | None | 1.189127973 | 6.463878327 |
