# Supplementary material for: Detailed comparison of two popular variant calling packages for exome and targeted exon studies
Source: PeerJ. 2014 Sep 30;2:e600. doi: 10.7717/peerj.600 (PMC4184249; doi:10.7717/peerj.600)
Supplement: Table S6 [file peerj-02-600-s025.doc]

**Table S6: 1KG Exome Recovery of 454 PCR Validated SNPs**

| **Variant.Caller** | **Preprocessing** | **SubjectID** | **Pos** | **hg19.Ref** | **hg19.Var** | **1KG.Ref** | **1KG.Var** | **Concordance** |
| --- | --- | --- | --- | --- | --- | --- | --- | --- |
| GATK.Haplotype.all | Base.Recalibration | NA11893 | 34611605 | C | T | C | T | Yes |
| GATK.Haplotype.all | Full.Pipeline | NA11893 | 34611605 | C | T | C | T | Yes |
| GATK.Haplotype.all | Indel.Realignment | NA11893 | 34611605 | C | T | C | T | Yes |
| GATK.Haplotype.all | None | NA11893 | 34611605 | C | T | C | T | Yes |
| GATK.Haplotype.HQ | Base.Recalibration | NA11893 | 34611605 | C | T | C | T | Yes |
| GATK.Haplotype.HQ | Full.Pipeline | NA11893 | 34611605 | C | T | C | T | Yes |
| GATK.Haplotype.HQ | Indel.Realignment | NA11893 | 34611605 | C | T | C | T | Yes |
| GATK.Haplotype.HQ | None | NA11893 | 34611605 | C | T | C | T | Yes |
| GATK.Unified.all | Base.Recalibration | NA11893 | 34611605 | C | T | C | T | Yes |
| GATK.Unified.all | Full.Pipeline | NA11893 | 34611605 | C | T | C | T | Yes |
| GATK.Unified.all | Indel.Realignment | NA11893 | 34611605 | C | T | C | T | Yes |
| GATK.Unified.all | None | NA11893 | 34611605 | C | T | C | T | Yes |
| GATK.Unified.HQ | Base.Recalibration | NA11893 | 34611605 | C | T | C | T | Yes |
| GATK.Unified.HQ | Full.Pipeline | NA11893 | 34611605 | C | T | C | T | Yes |
| GATK.Unified.HQ | Indel.Realignment | NA11893 | 34611605 | C | T | C | T | Yes |
| GATK.Unified.HQ | None | NA11893 | 34611605 | C | T | C | T | Yes |
| VarScan | Base.Recalibration | NA11893 | 34611605 | C | T | C | T | Yes |
| VarScan | Full.Pipeline | NA11893 | 34611605 | C | T | C | T | Yes |
| VarScan | Indel.Realignment | NA11893 | 34611605 | C | T | C | T | Yes |
| VarScan | None | NA11893 | 34611605 | C | T | C | T | Yes |
| VarScan.custom | Base.Recalibration | NA11893 | 34611605 | C | T | C | T | Yes |
| VarScan.custom | Full.Pipeline | NA11893 | 34611605 | C | T | C | T | Yes |
| VarScan.custom | Indel.Realignment | NA11893 | 34611605 | C | T | C | T | Yes |
| VarScan.custom | None | NA11893 | 34611605 | C | T | C | T | Yes |
| VarScan.pvalue | Base.Recalibration | NA11893 | 34611605 | C | T | C | T | Yes |
| VarScan.pvalue | Full.Pipeline | NA11893 | 34611605 | C | T | C | T | Yes |
| VarScan.pvalue | Indel.Realignment | NA11893 | 34611605 | C | T | C | T | Yes |
| VarScan.pvalue | None | NA11893 | 34611605 | C | T | C | T | Yes |
| GATK.Haplotype.all | Base.Recalibration | NA11893 | 36641871 | G | A | G | A | Yes |
| GATK.Haplotype.all | Full.Pipeline | NA11893 | 36641871 | G | A | G | A | Yes |
| GATK.Haplotype.all | Indel.Realignment | NA11893 | 36641871 | G | A | G | A | Yes |
| GATK.Haplotype.all | None | NA11893 | 36641871 | G | A | G | A | Yes |
| GATK.Haplotype.HQ | Base.Recalibration | NA11893 | 36641871 | G | A | G | A | Yes |
| GATK.Haplotype.HQ | Full.Pipeline | NA11893 | 36641871 | G | A | G | A | Yes |
| GATK.Haplotype.HQ | Indel.Realignment | NA11893 | 36641871 | G | A | G | A | Yes |
| GATK.Haplotype.HQ | None | NA11893 | 36641871 | G | A | G | A | Yes |
| GATK.Unified.all | Base.Recalibration | NA11893 | 36641871 | G | A | G | A | Yes |
| GATK.Unified.all | Full.Pipeline | NA11893 | 36641871 | G | A | G | A | Yes |
| GATK.Unified.all | Indel.Realignment | NA11893 | 36641871 | G | A | G | A | Yes |
| GATK.Unified.all | None | NA11893 | 36641871 | G | A | G | A | Yes |
| GATK.Unified.HQ | Base.Recalibration | NA11893 | 36641871 | G | A | G | A | Yes |
| GATK.Unified.HQ | Full.Pipeline | NA11893 | 36641871 | G | A | G | A | Yes |
| GATK.Unified.HQ | Indel.Realignment | NA11893 | 36641871 | G | A | G | A | Yes |
| GATK.Unified.HQ | None | NA11893 | 36641871 | G | A | G | A | Yes |
| VarScan | Base.Recalibration | NA11893 | 36641871 | G | A | G | A | Yes |
| VarScan | Full.Pipeline | NA11893 | 36641871 | G | A | G | A | Yes |
| VarScan | Indel.Realignment | NA11893 | 36641871 | G | A | G | A | Yes |
| VarScan | None | NA11893 | 36641871 | G | A | G | A | Yes |
| VarScan.custom | Base.Recalibration | NA11893 | 36641871 | G | A | G | A | Yes |
| VarScan.custom | Full.Pipeline | NA11893 | 36641871 | G | A | G | A | Yes |
| VarScan.custom | Indel.Realignment | NA11893 | 36641871 | G | A | G | A | Yes |
| VarScan.custom | None | NA11893 | 36641871 | G | A | G | A | Yes |
| VarScan.pvalue | Base.Recalibration | NA11893 | 36641871 | G | A | G | A | Yes |
| VarScan.pvalue | Full.Pipeline | NA11893 | 36641871 | G | A | G | A | Yes |
| VarScan.pvalue | Indel.Realignment | NA11893 | 36641871 | G | A | G | A | Yes |
| VarScan.pvalue | None | NA11893 | 36641871 | G | A | G | A | Yes |
| GATK.Haplotype.all | Base.Recalibration | NA12287 | 371958 | G | A | G | A | Yes |
| GATK.Haplotype.all | Full.Pipeline | NA12287 | 371958 | G | A | G | A | Yes |
| GATK.Haplotype.all | Indel.Realignment | NA12287 | 371958 | G | A | G | A | Yes |
| GATK.Haplotype.all | None | NA12287 | 371958 | G | A | G | A | Yes |
| GATK.Haplotype.HQ | Base.Recalibration | NA12287 | 371958 | G | A | G | A | Yes |
| GATK.Haplotype.HQ | Full.Pipeline | NA12287 | 371958 | G | A | G | A | Yes |
| GATK.Haplotype.HQ | Indel.Realignment | NA12287 | 371958 | G | A | G | A | Yes |
| GATK.Haplotype.HQ | None | NA12287 | 371958 | G | A | G | A | Yes |
| GATK.Unified.all | Base.Recalibration | NA12287 | 371958 | G | A | G | A | Yes |
| GATK.Unified.all | Full.Pipeline | NA12287 | 371958 | G | A | G | A | Yes |
| GATK.Unified.all | Indel.Realignment | NA12287 | 371958 | G | A | G | A | Yes |
| GATK.Unified.all | None | NA12287 | 371958 | G | A | G | A | Yes |
| GATK.Unified.HQ | Base.Recalibration | NA12287 | 371958 | G | A | G | A | Yes |
| GATK.Unified.HQ | Full.Pipeline | NA12287 | 371958 | G | A | G | A | Yes |
| GATK.Unified.HQ | Indel.Realignment | NA12287 | 371958 | G | A | G | A | Yes |
| GATK.Unified.HQ | None | NA12287 | 371958 | G | A | G | A | Yes |
| VarScan | Base.Recalibration | NA12287 | 371958 | G | A | G | A | Yes |
| VarScan | Full.Pipeline | NA12287 | 371958 | G | A | G | A | Yes |
| VarScan | Indel.Realignment | NA12287 | 371958 | G | A | G | A | Yes |
| VarScan | None | NA12287 | 371958 | G | A | G | A | Yes |
| VarScan.custom | Base.Recalibration | NA12287 | 371958 | G | A | NA | NA | **No** |
| VarScan.custom | Full.Pipeline | NA12287 | 371958 | G | A | NA | NA | **No** |
| VarScan.custom | Indel.Realignment | NA12287 | 371958 | G | A | NA | NA | **No** |
| VarScan.custom | None | NA12287 | 371958 | G | A | NA | NA | **No** |
| VarScan.pvalue | Base.Recalibration | NA12287 | 371958 | G | A | NA | NA | **No** |
| VarScan.pvalue | Full.Pipeline | NA12287 | 371958 | G | A | NA | NA | **No** |
| VarScan.pvalue | Indel.Realignment | NA12287 | 371958 | G | A | NA | NA | **No** |
| VarScan.pvalue | None | NA12287 | 371958 | G | A | NA | NA | **No** |
| GATK.Haplotype.all | Base.Recalibration | NA12287 | 30407978 | A | G | A | G | Yes |
| GATK.Haplotype.all | Full.Pipeline | NA12287 | 30407978 | A | G | A | G | Yes |
| GATK.Haplotype.all | Indel.Realignment | NA12287 | 30407978 | A | G | A | G | Yes |
| GATK.Haplotype.all | None | NA12287 | 30407978 | A | G | A | G | Yes |
| GATK.Haplotype.HQ | Base.Recalibration | NA12287 | 30407978 | A | G | A | G | Yes |
| GATK.Haplotype.HQ | Full.Pipeline | NA12287 | 30407978 | A | G | A | G | Yes |
| GATK.Haplotype.HQ | Indel.Realignment | NA12287 | 30407978 | A | G | A | G | Yes |
| GATK.Haplotype.HQ | None | NA12287 | 30407978 | A | G | A | G | Yes |
| GATK.Unified.all | Base.Recalibration | NA12287 | 30407978 | A | G | A | G | Yes |
| GATK.Unified.all | Full.Pipeline | NA12287 | 30407978 | A | G | A | G | Yes |
| GATK.Unified.all | Indel.Realignment | NA12287 | 30407978 | A | G | A | G | Yes |
| GATK.Unified.all | None | NA12287 | 30407978 | A | G | A | G | Yes |
| GATK.Unified.HQ | Base.Recalibration | NA12287 | 30407978 | A | G | A | G | Yes |
| GATK.Unified.HQ | Full.Pipeline | NA12287 | 30407978 | A | G | A | G | Yes |
| GATK.Unified.HQ | Indel.Realignment | NA12287 | 30407978 | A | G | A | G | Yes |
| GATK.Unified.HQ | None | NA12287 | 30407978 | A | G | A | G | Yes |
| VarScan | Base.Recalibration | NA12287 | 30407978 | A | G | A | G | Yes |
| VarScan | Full.Pipeline | NA12287 | 30407978 | A | G | A | G | Yes |
| VarScan | Indel.Realignment | NA12287 | 30407978 | A | G | A | G | Yes |
| VarScan | None | NA12287 | 30407978 | A | G | A | G | Yes |
| VarScan.custom | Base.Recalibration | NA12287 | 30407978 | A | G | A | G | Yes |
| VarScan.custom | Full.Pipeline | NA12287 | 30407978 | A | G | A | G | Yes |
| VarScan.custom | Indel.Realignment | NA12287 | 30407978 | A | G | A | G | Yes |
| VarScan.custom | None | NA12287 | 30407978 | A | G | A | G | Yes |
| VarScan.pvalue | Base.Recalibration | NA12287 | 30407978 | A | G | A | G | Yes |
| VarScan.pvalue | Full.Pipeline | NA12287 | 30407978 | A | G | A | G | Yes |
| VarScan.pvalue | Indel.Realignment | NA12287 | 30407978 | A | G | A | G | Yes |
| VarScan.pvalue | None | NA12287 | 30407978 | A | G | A | G | Yes |
| GATK.Haplotype.all | Base.Recalibration | NA18505 | 947909 | C | T | C | T | Yes |
| GATK.Haplotype.all | Full.Pipeline | NA18505 | 947909 | C | T | C | T | Yes |
| GATK.Haplotype.all | Indel.Realignment | NA18505 | 947909 | C | T | C | T | Yes |
| GATK.Haplotype.all | None | NA18505 | 947909 | C | T | C | T | Yes |
| GATK.Haplotype.HQ | Base.Recalibration | NA18505 | 947909 | C | T | C | T | Yes |
| GATK.Haplotype.HQ | Full.Pipeline | NA18505 | 947909 | C | T | C | T | Yes |
| GATK.Haplotype.HQ | Indel.Realignment | NA18505 | 947909 | C | T | C | T | Yes |
| GATK.Haplotype.HQ | None | NA18505 | 947909 | C | T | C | T | Yes |
| GATK.Unified.all | Base.Recalibration | NA18505 | 947909 | C | T | C | T | Yes |
| GATK.Unified.all | Full.Pipeline | NA18505 | 947909 | C | T | C | T | Yes |
| GATK.Unified.all | Indel.Realignment | NA18505 | 947909 | C | T | C | T | Yes |
| GATK.Unified.all | None | NA18505 | 947909 | C | T | C | T | Yes |
| GATK.Unified.HQ | Base.Recalibration | NA18505 | 947909 | C | T | C | T | Yes |
| GATK.Unified.HQ | Full.Pipeline | NA18505 | 947909 | C | T | C | T | Yes |
| GATK.Unified.HQ | Indel.Realignment | NA18505 | 947909 | C | T | C | T | Yes |
| GATK.Unified.HQ | None | NA18505 | 947909 | C | T | C | T | Yes |
| VarScan | Base.Recalibration | NA18505 | 947909 | C | T | C | T | Yes |
| VarScan | Full.Pipeline | NA18505 | 947909 | C | T | C | T | Yes |
| VarScan | Indel.Realignment | NA18505 | 947909 | C | T | C | T | Yes |
| VarScan | None | NA18505 | 947909 | C | T | C | T | Yes |
| VarScan.custom | Base.Recalibration | NA18505 | 947909 | C | T | C | T | Yes |
| VarScan.custom | Full.Pipeline | NA18505 | 947909 | C | T | C | T | Yes |
| VarScan.custom | Indel.Realignment | NA18505 | 947909 | C | T | C | T | Yes |
| VarScan.custom | None | NA18505 | 947909 | C | T | C | T | Yes |
| VarScan.pvalue | Base.Recalibration | NA18505 | 947909 | C | T | C | T | Yes |
| VarScan.pvalue | Full.Pipeline | NA18505 | 947909 | C | T | C | T | Yes |
| VarScan.pvalue | Indel.Realignment | NA18505 | 947909 | C | T | C | T | Yes |
| VarScan.pvalue | None | NA18505 | 947909 | C | T | C | T | Yes |
| GATK.Haplotype.all | Base.Recalibration | NA18505 | 3193991 | G | T | NA | NA | **No** |
| GATK.Haplotype.all | Full.Pipeline | NA18505 | 3193991 | G | T | NA | NA | **No** |
| GATK.Haplotype.all | Indel.Realignment | NA18505 | 3193991 | G | T | NA | NA | **No** |
| GATK.Haplotype.all | None | NA18505 | 3193991 | G | T | NA | NA | **No** |
| GATK.Haplotype.HQ | Base.Recalibration | NA18505 | 3193991 | G | T | NA | NA | **No** |
| GATK.Haplotype.HQ | Full.Pipeline | NA18505 | 3193991 | G | T | NA | NA | **No** |
| GATK.Haplotype.HQ | Indel.Realignment | NA18505 | 3193991 | G | T | NA | NA | **No** |
| GATK.Haplotype.HQ | None | NA18505 | 3193991 | G | T | NA | NA | **No** |
| GATK.Unified.all | Base.Recalibration | NA18505 | 3193991 | G | T | NA | NA | **No** |
| GATK.Unified.all | Full.Pipeline | NA18505 | 3193991 | G | T | NA | NA | **No** |
| GATK.Unified.all | Indel.Realignment | NA18505 | 3193991 | G | T | NA | NA | **No** |
| GATK.Unified.all | None | NA18505 | 3193991 | G | T | NA | NA | **No** |
| GATK.Unified.HQ | Base.Recalibration | NA18505 | 3193991 | G | T | NA | NA | **No** |
| GATK.Unified.HQ | Full.Pipeline | NA18505 | 3193991 | G | T | NA | NA | **No** |
| GATK.Unified.HQ | Indel.Realignment | NA18505 | 3193991 | G | T | NA | NA | **No** |
| GATK.Unified.HQ | None | NA18505 | 3193991 | G | T | NA | NA | **No** |
| VarScan | Base.Recalibration | NA18505 | 3193991 | G | T | NA | NA | **No** |
| VarScan | Full.Pipeline | NA18505 | 3193991 | G | T | NA | NA | **No** |
| VarScan | Indel.Realignment | NA18505 | 3193991 | G | T | NA | NA | **No** |
| VarScan | None | NA18505 | 3193991 | G | T | NA | NA | **No** |
| VarScan.custom | Base.Recalibration | NA18505 | 3193991 | G | T | NA | NA | **No** |
| VarScan.custom | Full.Pipeline | NA18505 | 3193991 | G | T | NA | NA | **No** |
| VarScan.custom | Indel.Realignment | NA18505 | 3193991 | G | T | NA | NA | **No** |
| VarScan.custom | None | NA18505 | 3193991 | G | T | NA | NA | **No** |
| VarScan.pvalue | Base.Recalibration | NA18505 | 3193991 | G | T | NA | NA | **No** |
| VarScan.pvalue | Full.Pipeline | NA18505 | 3193991 | G | T | NA | NA | **No** |
| VarScan.pvalue | Indel.Realignment | NA18505 | 3193991 | G | T | NA | NA | **No** |
| VarScan.pvalue | None | NA18505 | 3193991 | G | T | NA | NA | **No** |
| GATK.Haplotype.all | Base.Recalibration | NA18505 | 21346241 | C | T | C | T | Yes |
| GATK.Haplotype.all | Full.Pipeline | NA18505 | 21346241 | C | T | C | T | Yes |
| GATK.Haplotype.all | Indel.Realignment | NA18505 | 21346241 | C | T | C | T | Yes |
| GATK.Haplotype.all | None | NA18505 | 21346241 | C | T | C | T | Yes |
| GATK.Haplotype.HQ | Base.Recalibration | NA18505 | 21346241 | C | T | C | T | Yes |
| GATK.Haplotype.HQ | Full.Pipeline | NA18505 | 21346241 | C | T | C | T | Yes |
| GATK.Haplotype.HQ | Indel.Realignment | NA18505 | 21346241 | C | T | C | T | Yes |
| GATK.Haplotype.HQ | None | NA18505 | 21346241 | C | T | C | T | Yes |
| GATK.Unified.all | Base.Recalibration | NA18505 | 21346241 | C | T | C | T | Yes |
| GATK.Unified.all | Full.Pipeline | NA18505 | 21346241 | C | T | C | T | Yes |
| GATK.Unified.all | Indel.Realignment | NA18505 | 21346241 | C | T | C | T | Yes |
| GATK.Unified.all | None | NA18505 | 21346241 | C | T | C | T | Yes |
| GATK.Unified.HQ | Base.Recalibration | NA18505 | 21346241 | C | T | C | T | Yes |
| GATK.Unified.HQ | Full.Pipeline | NA18505 | 21346241 | C | T | C | T | Yes |
| GATK.Unified.HQ | Indel.Realignment | NA18505 | 21346241 | C | T | C | T | Yes |
| GATK.Unified.HQ | None | NA18505 | 21346241 | C | T | C | T | Yes |
| VarScan | Base.Recalibration | NA18505 | 21346241 | C | T | C | T | Yes |
| VarScan | Full.Pipeline | NA18505 | 21346241 | C | T | C | T | Yes |
| VarScan | Indel.Realignment | NA18505 | 21346241 | C | T | C | T | Yes |
| VarScan | None | NA18505 | 21346241 | C | T | C | T | Yes |
| VarScan.custom | Base.Recalibration | NA18505 | 21346241 | C | T | C | T | Yes |
| VarScan.custom | Full.Pipeline | NA18505 | 21346241 | C | T | C | T | Yes |
| VarScan.custom | Indel.Realignment | NA18505 | 21346241 | C | T | C | T | Yes |
| VarScan.custom | None | NA18505 | 21346241 | C | T | C | T | Yes |
| VarScan.pvalue | Base.Recalibration | NA18505 | 21346241 | C | T | C | T | Yes |
| VarScan.pvalue | Full.Pipeline | NA18505 | 21346241 | C | T | C | T | Yes |
| VarScan.pvalue | Indel.Realignment | NA18505 | 21346241 | C | T | C | T | Yes |
| VarScan.pvalue | None | NA18505 | 21346241 | C | T | C | T | Yes |
| GATK.Haplotype.all | Base.Recalibration | NA18505 | 39993724 | C | T | C | T | Yes |
| GATK.Haplotype.all | Full.Pipeline | NA18505 | 39993724 | C | T | C | T | Yes |
| GATK.Haplotype.all | Indel.Realignment | NA18505 | 39993724 | C | T | C | T | Yes |
| GATK.Haplotype.all | None | NA18505 | 39993724 | C | T | C | T | Yes |
| GATK.Haplotype.HQ | Base.Recalibration | NA18505 | 39993724 | C | T | C | T | Yes |
| GATK.Haplotype.HQ | Full.Pipeline | NA18505 | 39993724 | C | T | C | T | Yes |
| GATK.Haplotype.HQ | Indel.Realignment | NA18505 | 39993724 | C | T | C | T | Yes |
| GATK.Haplotype.HQ | None | NA18505 | 39993724 | C | T | C | T | Yes |
| GATK.Unified.all | Base.Recalibration | NA18505 | 39993724 | C | T | C | T | Yes |
| GATK.Unified.all | Full.Pipeline | NA18505 | 39993724 | C | T | C | T | Yes |
| GATK.Unified.all | Indel.Realignment | NA18505 | 39993724 | C | T | C | T | Yes |
| GATK.Unified.all | None | NA18505 | 39993724 | C | T | C | T | Yes |
| GATK.Unified.HQ | Base.Recalibration | NA18505 | 39993724 | C | T | C | T | Yes |
| GATK.Unified.HQ | Full.Pipeline | NA18505 | 39993724 | C | T | C | T | Yes |
| GATK.Unified.HQ | Indel.Realignment | NA18505 | 39993724 | C | T | C | T | Yes |
| GATK.Unified.HQ | None | NA18505 | 39993724 | C | T | C | T | Yes |
| VarScan | Base.Recalibration | NA18505 | 39993724 | C | T | C | T | Yes |
| VarScan | Full.Pipeline | NA18505 | 39993724 | C | T | C | T | Yes |
| VarScan | Indel.Realignment | NA18505 | 39993724 | C | T | C | T | Yes |
| VarScan | None | NA18505 | 39993724 | C | T | C | T | Yes |
| VarScan.custom | Base.Recalibration | NA18505 | 39993724 | C | T | C | T | Yes |
| VarScan.custom | Full.Pipeline | NA18505 | 39993724 | C | T | C | T | Yes |
| VarScan.custom | Indel.Realignment | NA18505 | 39993724 | C | T | C | T | Yes |
| VarScan.custom | None | NA18505 | 39993724 | C | T | C | T | Yes |
| VarScan.pvalue | Base.Recalibration | NA18505 | 39993724 | C | T | C | T | Yes |
| VarScan.pvalue | Full.Pipeline | NA18505 | 39993724 | C | T | C | T | Yes |
| VarScan.pvalue | Indel.Realignment | NA18505 | 39993724 | C | T | C | T | Yes |
| VarScan.pvalue | None | NA18505 | 39993724 | C | T | C | T | Yes |
| GATK.Haplotype.all | Base.Recalibration | NA18510 | 1145000 | C | T | C | T | Yes |
| GATK.Haplotype.all | Full.Pipeline | NA18510 | 1145000 | C | T | C | T | Yes |
| GATK.Haplotype.all | Indel.Realignment | NA18510 | 1145000 | C | T | C | T | Yes |
| GATK.Haplotype.all | None | NA18510 | 1145000 | C | T | C | T | Yes |
| GATK.Haplotype.HQ | Base.Recalibration | NA18510 | 1145000 | C | T | C | T | Yes |
| GATK.Haplotype.HQ | Full.Pipeline | NA18510 | 1145000 | C | T | C | T | Yes |
| GATK.Haplotype.HQ | Indel.Realignment | NA18510 | 1145000 | C | T | C | T | Yes |
| GATK.Haplotype.HQ | None | NA18510 | 1145000 | C | T | C | T | Yes |
| GATK.Unified.all | Base.Recalibration | NA18510 | 1145000 | C | T | C | T | Yes |
| GATK.Unified.all | Full.Pipeline | NA18510 | 1145000 | C | T | C | T | Yes |
| GATK.Unified.all | Indel.Realignment | NA18510 | 1145000 | C | T | C | T | Yes |
| GATK.Unified.all | None | NA18510 | 1145000 | C | T | C | T | Yes |
| GATK.Unified.HQ | Base.Recalibration | NA18510 | 1145000 | C | T | C | T | Yes |
| GATK.Unified.HQ | Full.Pipeline | NA18510 | 1145000 | C | T | C | T | Yes |
| GATK.Unified.HQ | Indel.Realignment | NA18510 | 1145000 | C | T | C | T | Yes |
| GATK.Unified.HQ | None | NA18510 | 1145000 | C | T | C | T | Yes |
| VarScan | Base.Recalibration | NA18510 | 1145000 | C | T | C | T | Yes |
| VarScan | Full.Pipeline | NA18510 | 1145000 | C | T | C | T | Yes |
| VarScan | Indel.Realignment | NA18510 | 1145000 | C | T | C | T | Yes |
| VarScan | None | NA18510 | 1145000 | C | T | C | T | Yes |
| VarScan.custom | Base.Recalibration | NA18510 | 1145000 | C | T | C | T | Yes |
| VarScan.custom | Full.Pipeline | NA18510 | 1145000 | C | T | C | T | Yes |
| VarScan.custom | Indel.Realignment | NA18510 | 1145000 | C | T | C | T | Yes |
| VarScan.custom | None | NA18510 | 1145000 | C | T | C | T | Yes |
| VarScan.pvalue | Base.Recalibration | NA18510 | 1145000 | C | T | C | T | Yes |
| VarScan.pvalue | Full.Pipeline | NA18510 | 1145000 | C | T | C | T | Yes |
| VarScan.pvalue | Indel.Realignment | NA18510 | 1145000 | C | T | C | T | Yes |
| VarScan.pvalue | None | NA18510 | 1145000 | C | T | C | T | Yes |
| GATK.Haplotype.all | Base.Recalibration | NA18510 | 23667835 | A | C | NA | NA | **No** |
| GATK.Haplotype.all | Full.Pipeline | NA18510 | 23667835 | A | C | NA | NA | **No** |
| GATK.Haplotype.all | Indel.Realignment | NA18510 | 23667835 | A | C | NA | NA | **No** |
| GATK.Haplotype.all | None | NA18510 | 23667835 | A | C | NA | NA | **No** |
| GATK.Haplotype.HQ | Base.Recalibration | NA18510 | 23667835 | A | C | NA | NA | **No** |
| GATK.Haplotype.HQ | Full.Pipeline | NA18510 | 23667835 | A | C | NA | NA | **No** |
| GATK.Haplotype.HQ | Indel.Realignment | NA18510 | 23667835 | A | C | NA | NA | **No** |
| GATK.Haplotype.HQ | None | NA18510 | 23667835 | A | C | NA | NA | **No** |
| GATK.Unified.all | Base.Recalibration | NA18510 | 23667835 | A | C | A | C | Yes |
| GATK.Unified.all | Full.Pipeline | NA18510 | 23667835 | A | C | A | C | Yes |
| GATK.Unified.all | Indel.Realignment | NA18510 | 23667835 | A | C | A | C | Yes |
| GATK.Unified.all | None | NA18510 | 23667835 | A | C | A | C | Yes |
| GATK.Unified.HQ | Base.Recalibration | NA18510 | 23667835 | A | C | NA | NA | **No** |
| GATK.Unified.HQ | Full.Pipeline | NA18510 | 23667835 | A | C | NA | NA | **No** |
| GATK.Unified.HQ | Indel.Realignment | NA18510 | 23667835 | A | C | A | C | Yes |
| GATK.Unified.HQ | None | NA18510 | 23667835 | A | C | A | C | Yes |
| VarScan | Base.Recalibration | NA18510 | 23667835 | A | C | A | C | Yes |
| VarScan | Full.Pipeline | NA18510 | 23667835 | A | C | A | C | Yes |
| VarScan | Indel.Realignment | NA18510 | 23667835 | A | C | A | C | Yes |
| VarScan | None | NA18510 | 23667835 | A | C | A | C | Yes |
| VarScan.custom | Base.Recalibration | NA18510 | 23667835 | A | C | NA | NA | **No** |
| VarScan.custom | Full.Pipeline | NA18510 | 23667835 | A | C | NA | NA | **No** |
| VarScan.custom | Indel.Realignment | NA18510 | 23667835 | A | C | NA | NA | **No** |
| VarScan.custom | None | NA18510 | 23667835 | A | C | NA | NA | **No** |
| VarScan.pvalue | Base.Recalibration | NA18510 | 23667835 | A | C | A | C | Yes |
| VarScan.pvalue | Full.Pipeline | NA18510 | 23667835 | A | C | A | C | Yes |
| VarScan.pvalue | Indel.Realignment | NA18510 | 23667835 | A | C | A | C | Yes |
| VarScan.pvalue | None | NA18510 | 23667835 | A | C | A | C | Yes |
| GATK.Haplotype.all | Base.Recalibration | NA18510 | 30432571 | T | C | T | C | Yes |
| GATK.Haplotype.all | Full.Pipeline | NA18510 | 30432571 | T | C | T | C | Yes |
| GATK.Haplotype.all | Indel.Realignment | NA18510 | 30432571 | T | C | T | C | Yes |
| GATK.Haplotype.all | None | NA18510 | 30432571 | T | C | T | C | Yes |
| GATK.Haplotype.HQ | Base.Recalibration | NA18510 | 30432571 | T | C | T | C | Yes |
| GATK.Haplotype.HQ | Full.Pipeline | NA18510 | 30432571 | T | C | T | C | Yes |
| GATK.Haplotype.HQ | Indel.Realignment | NA18510 | 30432571 | T | C | T | C | Yes |
| GATK.Haplotype.HQ | None | NA18510 | 30432571 | T | C | T | C | Yes |
| GATK.Unified.all | Base.Recalibration | NA18510 | 30432571 | T | C | T | C | Yes |
| GATK.Unified.all | Full.Pipeline | NA18510 | 30432571 | T | C | T | C | Yes |
| GATK.Unified.all | Indel.Realignment | NA18510 | 30432571 | T | C | T | C | Yes |
| GATK.Unified.all | None | NA18510 | 30432571 | T | C | T | C | Yes |
| GATK.Unified.HQ | Base.Recalibration | NA18510 | 30432571 | T | C | T | C | Yes |
| GATK.Unified.HQ | Full.Pipeline | NA18510 | 30432571 | T | C | T | C | Yes |
| GATK.Unified.HQ | Indel.Realignment | NA18510 | 30432571 | T | C | T | C | Yes |
| GATK.Unified.HQ | None | NA18510 | 30432571 | T | C | T | C | Yes |
| VarScan | Base.Recalibration | NA18510 | 30432571 | T | C | T | C | Yes |
| VarScan | Full.Pipeline | NA18510 | 30432571 | T | C | T | C | Yes |
| VarScan | Indel.Realignment | NA18510 | 30432571 | T | C | T | C | Yes |
| VarScan | None | NA18510 | 30432571 | T | C | T | C | Yes |
| VarScan.custom | Base.Recalibration | NA18510 | 30432571 | T | C | T | C | Yes |
| VarScan.custom | Full.Pipeline | NA18510 | 30432571 | T | C | T | C | Yes |
| VarScan.custom | Indel.Realignment | NA18510 | 30432571 | T | C | T | C | Yes |
| VarScan.custom | None | NA18510 | 30432571 | T | C | T | C | Yes |
| VarScan.pvalue | Base.Recalibration | NA18510 | 30432571 | T | C | T | C | Yes |
| VarScan.pvalue | Full.Pipeline | NA18510 | 30432571 | T | C | T | C | Yes |
| VarScan.pvalue | Indel.Realignment | NA18510 | 30432571 | T | C | T | C | Yes |
| VarScan.pvalue | None | NA18510 | 30432571 | T | C | T | C | Yes |
| GATK.Haplotype.all | Base.Recalibration | NA18510 | 32298534 | G | A | G | A | Yes |
| GATK.Haplotype.all | Full.Pipeline | NA18510 | 32298534 | G | A | G | A | Yes |
| GATK.Haplotype.all | Indel.Realignment | NA18510 | 32298534 | G | A | G | A | Yes |
| GATK.Haplotype.all | None | NA18510 | 32298534 | G | A | G | A | Yes |
| GATK.Haplotype.HQ | Base.Recalibration | NA18510 | 32298534 | G | A | G | A | Yes |
| GATK.Haplotype.HQ | Full.Pipeline | NA18510 | 32298534 | G | A | G | A | Yes |
| GATK.Haplotype.HQ | Indel.Realignment | NA18510 | 32298534 | G | A | G | A | Yes |
| GATK.Haplotype.HQ | None | NA18510 | 32298534 | G | A | G | A | Yes |
| GATK.Unified.all | Base.Recalibration | NA18510 | 32298534 | G | A | G | A | Yes |
| GATK.Unified.all | Full.Pipeline | NA18510 | 32298534 | G | A | G | A | Yes |
| GATK.Unified.all | Indel.Realignment | NA18510 | 32298534 | G | A | G | A | Yes |
| GATK.Unified.all | None | NA18510 | 32298534 | G | A | G | A | Yes |
| GATK.Unified.HQ | Base.Recalibration | NA18510 | 32298534 | G | A | G | A | Yes |
| GATK.Unified.HQ | Full.Pipeline | NA18510 | 32298534 | G | A | G | A | Yes |
| GATK.Unified.HQ | Indel.Realignment | NA18510 | 32298534 | G | A | G | A | Yes |
| GATK.Unified.HQ | None | NA18510 | 32298534 | G | A | G | A | Yes |
| VarScan | Base.Recalibration | NA18510 | 32298534 | G | A | G | A | Yes |
| VarScan | Full.Pipeline | NA18510 | 32298534 | G | A | G | A | Yes |
| VarScan | Indel.Realignment | NA18510 | 32298534 | G | A | G | A | Yes |
| VarScan | None | NA18510 | 32298534 | G | A | G | A | Yes |
| VarScan.custom | Base.Recalibration | NA18510 | 32298534 | G | A | G | A | Yes |
| VarScan.custom | Full.Pipeline | NA18510 | 32298534 | G | A | G | A | Yes |
| VarScan.custom | Indel.Realignment | NA18510 | 32298534 | G | A | G | A | Yes |
| VarScan.custom | None | NA18510 | 32298534 | G | A | G | A | Yes |
| VarScan.pvalue | Base.Recalibration | NA18510 | 32298534 | G | A | G | A | Yes |
| VarScan.pvalue | Full.Pipeline | NA18510 | 32298534 | G | A | G | A | Yes |
| VarScan.pvalue | Indel.Realignment | NA18510 | 32298534 | G | A | G | A | Yes |
| VarScan.pvalue | None | NA18510 | 32298534 | G | A | G | A | Yes |
| GATK.Haplotype.all | Base.Recalibration | NA18532 | 3687330 | T | C | T | C | Yes |
| GATK.Haplotype.all | Full.Pipeline | NA18532 | 3687330 | T | C | T | C | Yes |
| GATK.Haplotype.all | Indel.Realignment | NA18532 | 3687330 | T | C | T | C | Yes |
| GATK.Haplotype.all | None | NA18532 | 3687330 | T | C | T | C | Yes |
| GATK.Haplotype.HQ | Base.Recalibration | NA18532 | 3687330 | T | C | T | C | Yes |
| GATK.Haplotype.HQ | Full.Pipeline | NA18532 | 3687330 | T | C | T | C | Yes |
| GATK.Haplotype.HQ | Indel.Realignment | NA18532 | 3687330 | T | C | T | C | Yes |
| GATK.Haplotype.HQ | None | NA18532 | 3687330 | T | C | T | C | Yes |
| GATK.Unified.all | Base.Recalibration | NA18532 | 3687330 | T | C | T | C | Yes |
| GATK.Unified.all | Full.Pipeline | NA18532 | 3687330 | T | C | T | C | Yes |
| GATK.Unified.all | Indel.Realignment | NA18532 | 3687330 | T | C | T | C | Yes |
| GATK.Unified.all | None | NA18532 | 3687330 | T | C | T | C | Yes |
| GATK.Unified.HQ | Base.Recalibration | NA18532 | 3687330 | T | C | T | C | Yes |
| GATK.Unified.HQ | Full.Pipeline | NA18532 | 3687330 | T | C | T | C | Yes |
| GATK.Unified.HQ | Indel.Realignment | NA18532 | 3687330 | T | C | T | C | Yes |
| GATK.Unified.HQ | None | NA18532 | 3687330 | T | C | T | C | Yes |
| VarScan | Base.Recalibration | NA18532 | 3687330 | T | C | T | C | Yes |
| VarScan | Full.Pipeline | NA18532 | 3687330 | T | C | T | C | Yes |
| VarScan | Indel.Realignment | NA18532 | 3687330 | T | C | T | C | Yes |
| VarScan | None | NA18532 | 3687330 | T | C | T | C | Yes |
| VarScan.custom | Base.Recalibration | NA18532 | 3687330 | T | C | NA | NA | **No** |
| VarScan.custom | Full.Pipeline | NA18532 | 3687330 | T | C | NA | NA | **No** |
| VarScan.custom | Indel.Realignment | NA18532 | 3687330 | T | C | NA | NA | **No** |
| VarScan.custom | None | NA18532 | 3687330 | T | C | NA | NA | **No** |
| VarScan.pvalue | Base.Recalibration | NA18532 | 3687330 | T | C | T | C | Yes |
| VarScan.pvalue | Full.Pipeline | NA18532 | 3687330 | T | C | T | C | Yes |
| VarScan.pvalue | Indel.Realignment | NA18532 | 3687330 | T | C | T | C | Yes |
| VarScan.pvalue | None | NA18532 | 3687330 | T | C | T | C | Yes |
| GATK.Haplotype.all | Base.Recalibration | NA18532 | 32336755 | G | A | G | A | Yes |
| GATK.Haplotype.all | Full.Pipeline | NA18532 | 32336755 | G | A | G | A | Yes |
| GATK.Haplotype.all | Indel.Realignment | NA18532 | 32336755 | G | A | G | A | Yes |
| GATK.Haplotype.all | None | NA18532 | 32336755 | G | A | G | A | Yes |
| GATK.Haplotype.HQ | Base.Recalibration | NA18532 | 32336755 | G | A | G | A | Yes |
| GATK.Haplotype.HQ | Full.Pipeline | NA18532 | 32336755 | G | A | G | A | Yes |
| GATK.Haplotype.HQ | Indel.Realignment | NA18532 | 32336755 | G | A | G | A | Yes |
| GATK.Haplotype.HQ | None | NA18532 | 32336755 | G | A | G | A | Yes |
| GATK.Unified.all | Base.Recalibration | NA18532 | 32336755 | G | A | G | A | Yes |
| GATK.Unified.all | Full.Pipeline | NA18532 | 32336755 | G | A | G | A | Yes |
| GATK.Unified.all | Indel.Realignment | NA18532 | 32336755 | G | A | G | A | Yes |
| GATK.Unified.all | None | NA18532 | 32336755 | G | A | G | A | Yes |
| GATK.Unified.HQ | Base.Recalibration | NA18532 | 32336755 | G | A | G | A | Yes |
| GATK.Unified.HQ | Full.Pipeline | NA18532 | 32336755 | G | A | G | A | Yes |
| GATK.Unified.HQ | Indel.Realignment | NA18532 | 32336755 | G | A | G | A | Yes |
| GATK.Unified.HQ | None | NA18532 | 32336755 | G | A | G | A | Yes |
| VarScan | Base.Recalibration | NA18532 | 32336755 | G | A | G | A | Yes |
| VarScan | Full.Pipeline | NA18532 | 32336755 | G | A | G | A | Yes |
| VarScan | Indel.Realignment | NA18532 | 32336755 | G | A | G | A | Yes |
| VarScan | None | NA18532 | 32336755 | G | A | G | A | Yes |
| VarScan.custom | Base.Recalibration | NA18532 | 32336755 | G | A | G | A | Yes |
| VarScan.custom | Full.Pipeline | NA18532 | 32336755 | G | A | G | A | Yes |
| VarScan.custom | Indel.Realignment | NA18532 | 32336755 | G | A | G | A | Yes |
| VarScan.custom | None | NA18532 | 32336755 | G | A | G | A | Yes |
| VarScan.pvalue | Base.Recalibration | NA18532 | 32336755 | G | A | G | A | Yes |
| VarScan.pvalue | Full.Pipeline | NA18532 | 32336755 | G | A | G | A | Yes |
| VarScan.pvalue | Indel.Realignment | NA18532 | 32336755 | G | A | G | A | Yes |
| VarScan.pvalue | None | NA18532 | 32336755 | G | A | G | A | Yes |
| GATK.Haplotype.all | Base.Recalibration | NA18532 | 57769739 | A | G | NA | NA | **No** |
| GATK.Haplotype.all | Full.Pipeline | NA18532 | 57769739 | A | G | NA | NA | **No** |
| GATK.Haplotype.all | Indel.Realignment | NA18532 | 57769739 | A | G | NA | NA | **No** |
| GATK.Haplotype.all | None | NA18532 | 57769739 | A | G | NA | NA | **No** |
| GATK.Haplotype.HQ | Base.Recalibration | NA18532 | 57769739 | A | G | NA | NA | **No** |
| GATK.Haplotype.HQ | Full.Pipeline | NA18532 | 57769739 | A | G | NA | NA | **No** |
| GATK.Haplotype.HQ | Indel.Realignment | NA18532 | 57769739 | A | G | NA | NA | **No** |
| GATK.Haplotype.HQ | None | NA18532 | 57769739 | A | G | NA | NA | **No** |
| GATK.Unified.all | Base.Recalibration | NA18532 | 57769739 | A | G | NA | NA | **No** |
| GATK.Unified.all | Full.Pipeline | NA18532 | 57769739 | A | G | NA | NA | **No** |
| GATK.Unified.all | Indel.Realignment | NA18532 | 57769739 | A | G | NA | NA | **No** |
| GATK.Unified.all | None | NA18532 | 57769739 | A | G | NA | NA | **No** |
| GATK.Unified.HQ | Base.Recalibration | NA18532 | 57769739 | A | G | NA | NA | **No** |
| GATK.Unified.HQ | Full.Pipeline | NA18532 | 57769739 | A | G | NA | NA | **No** |
| GATK.Unified.HQ | Indel.Realignment | NA18532 | 57769739 | A | G | NA | NA | **No** |
| GATK.Unified.HQ | None | NA18532 | 57769739 | A | G | NA | NA | **No** |
| VarScan | Base.Recalibration | NA18532 | 57769739 | A | G | NA | NA | **No** |
| VarScan | Full.Pipeline | NA18532 | 57769739 | A | G | NA | NA | **No** |
| VarScan | Indel.Realignment | NA18532 | 57769739 | A | G | NA | NA | **No** |
| VarScan | None | NA18532 | 57769739 | A | G | NA | NA | **No** |
| VarScan.custom | Base.Recalibration | NA18532 | 57769739 | A | G | NA | NA | **No** |
| VarScan.custom | Full.Pipeline | NA18532 | 57769739 | A | G | NA | NA | **No** |
| VarScan.custom | Indel.Realignment | NA18532 | 57769739 | A | G | NA | NA | **No** |
| VarScan.custom | None | NA18532 | 57769739 | A | G | NA | NA | **No** |
| VarScan.pvalue | Base.Recalibration | NA18532 | 57769739 | A | G | NA | NA | **No** |
| VarScan.pvalue | Full.Pipeline | NA18532 | 57769739 | A | G | NA | NA | **No** |
| VarScan.pvalue | Indel.Realignment | NA18532 | 57769739 | A | G | NA | NA | **No** |
| VarScan.pvalue | None | NA18532 | 57769739 | A | G | NA | NA | **No** |
| GATK.Haplotype.all | Base.Recalibration | NA18566 | 23667794 | G | T | G | T | Yes |
| GATK.Haplotype.all | Full.Pipeline | NA18566 | 23667794 | G | T | G | T | Yes |
| GATK.Haplotype.all | Indel.Realignment | NA18566 | 23667794 | G | T | G | T | Yes |
| GATK.Haplotype.all | None | NA18566 | 23667794 | G | T | G | T | Yes |
| GATK.Haplotype.HQ | Base.Recalibration | NA18566 | 23667794 | G | T | G | T | Yes |
| GATK.Haplotype.HQ | Full.Pipeline | NA18566 | 23667794 | G | T | G | T | Yes |
| GATK.Haplotype.HQ | Indel.Realignment | NA18566 | 23667794 | G | T | G | T | Yes |
| GATK.Haplotype.HQ | None | NA18566 | 23667794 | G | T | G | T | Yes |
| GATK.Unified.all | Base.Recalibration | NA18566 | 23667794 | G | T | G | T | Yes |
| GATK.Unified.all | Full.Pipeline | NA18566 | 23667794 | G | T | G | T | Yes |
| GATK.Unified.all | Indel.Realignment | NA18566 | 23667794 | G | T | G | T | Yes |
| GATK.Unified.all | None | NA18566 | 23667794 | G | T | G | T | Yes |
| GATK.Unified.HQ | Base.Recalibration | NA18566 | 23667794 | G | T | G | T | Yes |
| GATK.Unified.HQ | Full.Pipeline | NA18566 | 23667794 | G | T | G | T | Yes |
| GATK.Unified.HQ | Indel.Realignment | NA18566 | 23667794 | G | T | G | T | Yes |
| GATK.Unified.HQ | None | NA18566 | 23667794 | G | T | G | T | Yes |
| VarScan | Base.Recalibration | NA18566 | 23667794 | G | T | G | T | Yes |
| VarScan | Full.Pipeline | NA18566 | 23667794 | G | T | G | T | Yes |
| VarScan | Indel.Realignment | NA18566 | 23667794 | G | T | G | T | Yes |
| VarScan | None | NA18566 | 23667794 | G | T | G | T | Yes |
| VarScan.custom | Base.Recalibration | NA18566 | 23667794 | G | T | G | T | Yes |
| VarScan.custom | Full.Pipeline | NA18566 | 23667794 | G | T | G | T | Yes |
| VarScan.custom | Indel.Realignment | NA18566 | 23667794 | G | T | G | T | Yes |
| VarScan.custom | None | NA18566 | 23667794 | G | T | G | T | Yes |
| VarScan.pvalue | Base.Recalibration | NA18566 | 23667794 | G | T | G | T | Yes |
| VarScan.pvalue | Full.Pipeline | NA18566 | 23667794 | G | T | G | T | Yes |
| VarScan.pvalue | Indel.Realignment | NA18566 | 23667794 | G | T | G | T | Yes |
| VarScan.pvalue | None | NA18566 | 23667794 | G | T | G | T | Yes |
| GATK.Haplotype.all | Base.Recalibration | NA18566 | 44580999 | T | C | T | C | Yes |
| GATK.Haplotype.all | Full.Pipeline | NA18566 | 44580999 | T | C | T | C | Yes |
| GATK.Haplotype.all | Indel.Realignment | NA18566 | 44580999 | T | C | T | C | Yes |
| GATK.Haplotype.all | None | NA18566 | 44580999 | T | C | T | C | Yes |
| GATK.Haplotype.HQ | Base.Recalibration | NA18566 | 44580999 | T | C | T | C | Yes |
| GATK.Haplotype.HQ | Full.Pipeline | NA18566 | 44580999 | T | C | T | C | Yes |
| GATK.Haplotype.HQ | Indel.Realignment | NA18566 | 44580999 | T | C | T | C | Yes |
| GATK.Haplotype.HQ | None | NA18566 | 44580999 | T | C | T | C | Yes |
| GATK.Unified.all | Base.Recalibration | NA18566 | 44580999 | T | C | T | C | Yes |
| GATK.Unified.all | Full.Pipeline | NA18566 | 44580999 | T | C | T | C | Yes |
| GATK.Unified.all | Indel.Realignment | NA18566 | 44580999 | T | C | T | C | Yes |
| GATK.Unified.all | None | NA18566 | 44580999 | T | C | T | C | Yes |
| GATK.Unified.HQ | Base.Recalibration | NA18566 | 44580999 | T | C | T | C | Yes |
| GATK.Unified.HQ | Full.Pipeline | NA18566 | 44580999 | T | C | T | C | Yes |
| GATK.Unified.HQ | Indel.Realignment | NA18566 | 44580999 | T | C | T | C | Yes |
| GATK.Unified.HQ | None | NA18566 | 44580999 | T | C | T | C | Yes |
| VarScan | Base.Recalibration | NA18566 | 44580999 | T | C | T | C | Yes |
| VarScan | Full.Pipeline | NA18566 | 44580999 | T | C | T | C | Yes |
| VarScan | Indel.Realignment | NA18566 | 44580999 | T | C | T | C | Yes |
| VarScan | None | NA18566 | 44580999 | T | C | T | C | Yes |
| VarScan.custom | Base.Recalibration | NA18566 | 44580999 | T | C | T | C | Yes |
| VarScan.custom | Full.Pipeline | NA18566 | 44580999 | T | C | T | C | Yes |
| VarScan.custom | Indel.Realignment | NA18566 | 44580999 | T | C | T | C | Yes |
| VarScan.custom | None | NA18566 | 44580999 | T | C | T | C | Yes |
| VarScan.pvalue | Base.Recalibration | NA18566 | 44580999 | T | C | T | C | Yes |
| VarScan.pvalue | Full.Pipeline | NA18566 | 44580999 | T | C | T | C | Yes |
| VarScan.pvalue | Indel.Realignment | NA18566 | 44580999 | T | C | T | C | Yes |
| VarScan.pvalue | None | NA18566 | 44580999 | T | C | T | C | Yes |
| GATK.Haplotype.all | Base.Recalibration | NA18637 | 25252067 | A | G | A | G | Yes |
| GATK.Haplotype.all | Full.Pipeline | NA18637 | 25252067 | A | G | A | G | Yes |
| GATK.Haplotype.all | Indel.Realignment | NA18637 | 25252067 | A | G | A | G | Yes |
| GATK.Haplotype.all | None | NA18637 | 25252067 | A | G | A | G | Yes |
| GATK.Haplotype.HQ | Base.Recalibration | NA18637 | 25252067 | A | G | A | G | Yes |
| GATK.Haplotype.HQ | Full.Pipeline | NA18637 | 25252067 | A | G | A | G | Yes |
| GATK.Haplotype.HQ | Indel.Realignment | NA18637 | 25252067 | A | G | A | G | Yes |
| GATK.Haplotype.HQ | None | NA18637 | 25252067 | A | G | A | G | Yes |
| GATK.Unified.all | Base.Recalibration | NA18637 | 25252067 | A | G | A | G | Yes |
| GATK.Unified.all | Full.Pipeline | NA18637 | 25252067 | A | G | A | G | Yes |
| GATK.Unified.all | Indel.Realignment | NA18637 | 25252067 | A | G | A | G | Yes |
| GATK.Unified.all | None | NA18637 | 25252067 | A | G | A | G | Yes |
| GATK.Unified.HQ | Base.Recalibration | NA18637 | 25252067 | A | G | A | G | Yes |
| GATK.Unified.HQ | Full.Pipeline | NA18637 | 25252067 | A | G | A | G | Yes |
| GATK.Unified.HQ | Indel.Realignment | NA18637 | 25252067 | A | G | A | G | Yes |
| GATK.Unified.HQ | None | NA18637 | 25252067 | A | G | A | G | Yes |
| VarScan | Base.Recalibration | NA18637 | 25252067 | A | G | A | G | Yes |
| VarScan | Full.Pipeline | NA18637 | 25252067 | A | G | A | G | Yes |
| VarScan | Indel.Realignment | NA18637 | 25252067 | A | G | A | G | Yes |
| VarScan | None | NA18637 | 25252067 | A | G | A | G | Yes |
| VarScan.custom | Base.Recalibration | NA18637 | 25252067 | A | G | A | G | Yes |
| VarScan.custom | Full.Pipeline | NA18637 | 25252067 | A | G | A | G | Yes |
| VarScan.custom | Indel.Realignment | NA18637 | 25252067 | A | G | A | G | Yes |
| VarScan.custom | None | NA18637 | 25252067 | A | G | A | G | Yes |
| VarScan.pvalue | Base.Recalibration | NA18637 | 25252067 | A | G | A | G | Yes |
| VarScan.pvalue | Full.Pipeline | NA18637 | 25252067 | A | G | A | G | Yes |
| VarScan.pvalue | Indel.Realignment | NA18637 | 25252067 | A | G | A | G | Yes |
| VarScan.pvalue | None | NA18637 | 25252067 | A | G | A | G | Yes |
| GATK.Haplotype.all | Base.Recalibration | NA18637 | 31024034 | G | A | G | A | Yes |
| GATK.Haplotype.all | Full.Pipeline | NA18637 | 31024034 | G | A | G | A | Yes |
| GATK.Haplotype.all | Indel.Realignment | NA18637 | 31024034 | G | A | G | A | Yes |
| GATK.Haplotype.all | None | NA18637 | 31024034 | G | A | G | A | Yes |
| GATK.Haplotype.HQ | Base.Recalibration | NA18637 | 31024034 | G | A | G | A | Yes |
| GATK.Haplotype.HQ | Full.Pipeline | NA18637 | 31024034 | G | A | G | A | Yes |
| GATK.Haplotype.HQ | Indel.Realignment | NA18637 | 31024034 | G | A | G | A | Yes |
| GATK.Haplotype.HQ | None | NA18637 | 31024034 | G | A | G | A | Yes |
| GATK.Unified.all | Base.Recalibration | NA18637 | 31024034 | G | A | G | A | Yes |
| GATK.Unified.all | Full.Pipeline | NA18637 | 31024034 | G | A | G | A | Yes |
| GATK.Unified.all | Indel.Realignment | NA18637 | 31024034 | G | A | G | A | Yes |
| GATK.Unified.all | None | NA18637 | 31024034 | G | A | G | A | Yes |
| GATK.Unified.HQ | Base.Recalibration | NA18637 | 31024034 | G | A | G | A | Yes |
| GATK.Unified.HQ | Full.Pipeline | NA18637 | 31024034 | G | A | G | A | Yes |
| GATK.Unified.HQ | Indel.Realignment | NA18637 | 31024034 | G | A | G | A | Yes |
| GATK.Unified.HQ | None | NA18637 | 31024034 | G | A | G | A | Yes |
| VarScan | Base.Recalibration | NA18637 | 31024034 | G | A | G | A | Yes |
| VarScan | Full.Pipeline | NA18637 | 31024034 | G | A | G | A | Yes |
| VarScan | Indel.Realignment | NA18637 | 31024034 | G | A | G | A | Yes |
| VarScan | None | NA18637 | 31024034 | G | A | G | A | Yes |
| VarScan.custom | Base.Recalibration | NA18637 | 31024034 | G | A | G | A | Yes |
| VarScan.custom | Full.Pipeline | NA18637 | 31024034 | G | A | G | A | Yes |
| VarScan.custom | Indel.Realignment | NA18637 | 31024034 | G | A | G | A | Yes |
| VarScan.custom | None | NA18637 | 31024034 | G | A | G | A | Yes |
| VarScan.pvalue | Base.Recalibration | NA18637 | 31024034 | G | A | G | A | Yes |
| VarScan.pvalue | Full.Pipeline | NA18637 | 31024034 | G | A | G | A | Yes |
| VarScan.pvalue | Indel.Realignment | NA18637 | 31024034 | G | A | G | A | Yes |
| VarScan.pvalue | None | NA18637 | 31024034 | G | A | G | A | Yes |
| GATK.Haplotype.all | Base.Recalibration | NA18637 | 50071137 | C | T | C | T | Yes |
| GATK.Haplotype.all | Full.Pipeline | NA18637 | 50071137 | C | T | C | T | Yes |
| GATK.Haplotype.all | Indel.Realignment | NA18637 | 50071137 | C | T | C | T | Yes |
| GATK.Haplotype.all | None | NA18637 | 50071137 | C | T | C | T | Yes |
| GATK.Haplotype.HQ | Base.Recalibration | NA18637 | 50071137 | C | T | C | T | Yes |
| GATK.Haplotype.HQ | Full.Pipeline | NA18637 | 50071137 | C | T | C | T | Yes |
| GATK.Haplotype.HQ | Indel.Realignment | NA18637 | 50071137 | C | T | C | T | Yes |
| GATK.Haplotype.HQ | None | NA18637 | 50071137 | C | T | C | T | Yes |
| GATK.Unified.all | Base.Recalibration | NA18637 | 50071137 | C | T | C | T | Yes |
| GATK.Unified.all | Full.Pipeline | NA18637 | 50071137 | C | T | C | T | Yes |
| GATK.Unified.all | Indel.Realignment | NA18637 | 50071137 | C | T | C | T | Yes |
| GATK.Unified.all | None | NA18637 | 50071137 | C | T | C | T | Yes |
| GATK.Unified.HQ | Base.Recalibration | NA18637 | 50071137 | C | T | C | T | Yes |
| GATK.Unified.HQ | Full.Pipeline | NA18637 | 50071137 | C | T | C | T | Yes |
| GATK.Unified.HQ | Indel.Realignment | NA18637 | 50071137 | C | T | C | T | Yes |
| GATK.Unified.HQ | None | NA18637 | 50071137 | C | T | C | T | Yes |
| VarScan | Base.Recalibration | NA18637 | 50071137 | C | T | C | T | Yes |
| VarScan | Full.Pipeline | NA18637 | 50071137 | C | T | C | T | Yes |
| VarScan | Indel.Realignment | NA18637 | 50071137 | C | T | C | T | Yes |
| VarScan | None | NA18637 | 50071137 | C | T | C | T | Yes |
| VarScan.custom | Base.Recalibration | NA18637 | 50071137 | C | T | C | T | Yes |
| VarScan.custom | Full.Pipeline | NA18637 | 50071137 | C | T | C | T | Yes |
| VarScan.custom | Indel.Realignment | NA18637 | 50071137 | C | T | C | T | Yes |
| VarScan.custom | None | NA18637 | 50071137 | C | T | C | T | Yes |
| VarScan.pvalue | Base.Recalibration | NA18637 | 50071137 | C | T | C | T | Yes |
| VarScan.pvalue | Full.Pipeline | NA18637 | 50071137 | C | T | C | T | Yes |
| VarScan.pvalue | Indel.Realignment | NA18637 | 50071137 | C | T | C | T | Yes |
| VarScan.pvalue | None | NA18637 | 50071137 | C | T | C | T | Yes |
| GATK.Haplotype.all | Base.Recalibration | NA18858 | 2969015 | C | G | C | G | Yes |
| GATK.Haplotype.all | Full.Pipeline | NA18858 | 2969015 | C | G | C | G | Yes |
| GATK.Haplotype.all | Indel.Realignment | NA18858 | 2969015 | C | G | C | G | Yes |
| GATK.Haplotype.all | None | NA18858 | 2969015 | C | G | C | G | Yes |
| GATK.Haplotype.HQ | Base.Recalibration | NA18858 | 2969015 | C | G | C | G | Yes |
| GATK.Haplotype.HQ | Full.Pipeline | NA18858 | 2969015 | C | G | C | G | Yes |
| GATK.Haplotype.HQ | Indel.Realignment | NA18858 | 2969015 | C | G | C | G | Yes |
| GATK.Haplotype.HQ | None | NA18858 | 2969015 | C | G | C | G | Yes |
| GATK.Unified.all | Base.Recalibration | NA18858 | 2969015 | C | G | C | G | Yes |
| GATK.Unified.all | Full.Pipeline | NA18858 | 2969015 | C | G | C | G | Yes |
| GATK.Unified.all | Indel.Realignment | NA18858 | 2969015 | C | G | C | G | Yes |
| GATK.Unified.all | None | NA18858 | 2969015 | C | G | C | G | Yes |
| GATK.Unified.HQ | Base.Recalibration | NA18858 | 2969015 | C | G | C | G | Yes |
| GATK.Unified.HQ | Full.Pipeline | NA18858 | 2969015 | C | G | C | G | Yes |
| GATK.Unified.HQ | Indel.Realignment | NA18858 | 2969015 | C | G | C | G | Yes |
| GATK.Unified.HQ | None | NA18858 | 2969015 | C | G | C | G | Yes |
| VarScan | Base.Recalibration | NA18858 | 2969015 | C | G | C | G | Yes |
| VarScan | Full.Pipeline | NA18858 | 2969015 | C | G | C | G | Yes |
| VarScan | Indel.Realignment | NA18858 | 2969015 | C | G | C | G | Yes |
| VarScan | None | NA18858 | 2969015 | C | G | C | G | Yes |
| VarScan.custom | Base.Recalibration | NA18858 | 2969015 | C | G | C | G | Yes |
| VarScan.custom | Full.Pipeline | NA18858 | 2969015 | C | G | C | G | Yes |
| VarScan.custom | Indel.Realignment | NA18858 | 2969015 | C | G | C | G | Yes |
| VarScan.custom | None | NA18858 | 2969015 | C | G | C | G | Yes |
| VarScan.pvalue | Base.Recalibration | NA18858 | 2969015 | C | G | C | G | Yes |
| VarScan.pvalue | Full.Pipeline | NA18858 | 2969015 | C | G | C | G | Yes |
| VarScan.pvalue | Indel.Realignment | NA18858 | 2969015 | C | G | C | G | Yes |
| VarScan.pvalue | None | NA18858 | 2969015 | C | G | C | G | Yes |
| GATK.Haplotype.all | Base.Recalibration | NA18858 | 9520133 | G | A | G | A | Yes |
| GATK.Haplotype.all | Full.Pipeline | NA18858 | 9520133 | G | A | G | A | Yes |
| GATK.Haplotype.all | Indel.Realignment | NA18858 | 9520133 | G | A | G | A | Yes |
| GATK.Haplotype.all | None | NA18858 | 9520133 | G | A | G | A | Yes |
| GATK.Haplotype.HQ | Base.Recalibration | NA18858 | 9520133 | G | A | G | A | Yes |
| GATK.Haplotype.HQ | Full.Pipeline | NA18858 | 9520133 | G | A | G | A | Yes |
| GATK.Haplotype.HQ | Indel.Realignment | NA18858 | 9520133 | G | A | G | A | Yes |
| GATK.Haplotype.HQ | None | NA18858 | 9520133 | G | A | G | A | Yes |
| GATK.Unified.all | Base.Recalibration | NA18858 | 9520133 | G | A | G | A | Yes |
| GATK.Unified.all | Full.Pipeline | NA18858 | 9520133 | G | A | G | A | Yes |
| GATK.Unified.all | Indel.Realignment | NA18858 | 9520133 | G | A | G | A | Yes |
| GATK.Unified.all | None | NA18858 | 9520133 | G | A | G | A | Yes |
| GATK.Unified.HQ | Base.Recalibration | NA18858 | 9520133 | G | A | G | A | Yes |
| GATK.Unified.HQ | Full.Pipeline | NA18858 | 9520133 | G | A | G | A | Yes |
| GATK.Unified.HQ | Indel.Realignment | NA18858 | 9520133 | G | A | G | A | Yes |
| GATK.Unified.HQ | None | NA18858 | 9520133 | G | A | G | A | Yes |
| VarScan | Base.Recalibration | NA18858 | 9520133 | G | A | G | A | Yes |
| VarScan | Full.Pipeline | NA18858 | 9520133 | G | A | G | A | Yes |
| VarScan | Indel.Realignment | NA18858 | 9520133 | G | A | G | A | Yes |
| VarScan | None | NA18858 | 9520133 | G | A | G | A | Yes |
| VarScan.custom | Base.Recalibration | NA18858 | 9520133 | G | A | G | A | Yes |
| VarScan.custom | Full.Pipeline | NA18858 | 9520133 | G | A | G | A | Yes |
| VarScan.custom | Indel.Realignment | NA18858 | 9520133 | G | A | G | A | Yes |
| VarScan.custom | None | NA18858 | 9520133 | G | A | G | A | Yes |
| VarScan.pvalue | Base.Recalibration | NA18858 | 9520133 | G | A | G | A | Yes |
| VarScan.pvalue | Full.Pipeline | NA18858 | 9520133 | G | A | G | A | Yes |
| VarScan.pvalue | Indel.Realignment | NA18858 | 9520133 | G | A | G | A | Yes |
| VarScan.pvalue | None | NA18858 | 9520133 | G | A | G | A | Yes |
| GATK.Haplotype.all | Base.Recalibration | NA18858 | 23667835 | A | C | A | C | Yes |
| GATK.Haplotype.all | Full.Pipeline | NA18858 | 23667835 | A | C | A | C | Yes |
| GATK.Haplotype.all | Indel.Realignment | NA18858 | 23667835 | A | C | A | C | Yes |
| GATK.Haplotype.all | None | NA18858 | 23667835 | A | C | A | C | Yes |
| GATK.Haplotype.HQ | Base.Recalibration | NA18858 | 23667835 | A | C | A | C | Yes |
| GATK.Haplotype.HQ | Full.Pipeline | NA18858 | 23667835 | A | C | A | C | Yes |
| GATK.Haplotype.HQ | Indel.Realignment | NA18858 | 23667835 | A | C | A | C | Yes |
| GATK.Haplotype.HQ | None | NA18858 | 23667835 | A | C | A | C | Yes |
| GATK.Unified.all | Base.Recalibration | NA18858 | 23667835 | A | C | A | C | Yes |
| GATK.Unified.all | Full.Pipeline | NA18858 | 23667835 | A | C | A | C | Yes |
| GATK.Unified.all | Indel.Realignment | NA18858 | 23667835 | A | C | A | C | Yes |
| GATK.Unified.all | None | NA18858 | 23667835 | A | C | A | C | Yes |
| GATK.Unified.HQ | Base.Recalibration | NA18858 | 23667835 | A | C | A | C | Yes |
| GATK.Unified.HQ | Full.Pipeline | NA18858 | 23667835 | A | C | A | C | Yes |
| GATK.Unified.HQ | Indel.Realignment | NA18858 | 23667835 | A | C | A | C | Yes |
| GATK.Unified.HQ | None | NA18858 | 23667835 | A | C | A | C | Yes |
| VarScan | Base.Recalibration | NA18858 | 23667835 | A | C | A | C | Yes |
| VarScan | Full.Pipeline | NA18858 | 23667835 | A | C | A | C | Yes |
| VarScan | Indel.Realignment | NA18858 | 23667835 | A | C | A | C | Yes |
| VarScan | None | NA18858 | 23667835 | A | C | A | C | Yes |
| VarScan.custom | Base.Recalibration | NA18858 | 23667835 | A | C | NA | NA | **No** |
| VarScan.custom | Full.Pipeline | NA18858 | 23667835 | A | C | NA | NA | **No** |
| VarScan.custom | Indel.Realignment | NA18858 | 23667835 | A | C | NA | NA | **No** |
| VarScan.custom | None | NA18858 | 23667835 | A | C | NA | NA | **No** |
| VarScan.pvalue | Base.Recalibration | NA18858 | 23667835 | A | C | A | C | Yes |
| VarScan.pvalue | Full.Pipeline | NA18858 | 23667835 | A | C | A | C | Yes |
| VarScan.pvalue | Indel.Realignment | NA18858 | 23667835 | A | C | A | C | Yes |
| VarScan.pvalue | None | NA18858 | 23667835 | A | C | A | C | Yes |
| GATK.Haplotype.all | Base.Recalibration | NA18858 | 30432571 | T | C | T | C | Yes |
| GATK.Haplotype.all | Full.Pipeline | NA18858 | 30432571 | T | C | T | C | Yes |
| GATK.Haplotype.all | Indel.Realignment | NA18858 | 30432571 | T | C | T | C | Yes |
| GATK.Haplotype.all | None | NA18858 | 30432571 | T | C | T | C | Yes |
| GATK.Haplotype.HQ | Base.Recalibration | NA18858 | 30432571 | T | C | T | C | Yes |
| GATK.Haplotype.HQ | Full.Pipeline | NA18858 | 30432571 | T | C | T | C | Yes |
| GATK.Haplotype.HQ | Indel.Realignment | NA18858 | 30432571 | T | C | T | C | Yes |
| GATK.Haplotype.HQ | None | NA18858 | 30432571 | T | C | T | C | Yes |
| GATK.Unified.all | Base.Recalibration | NA18858 | 30432571 | T | C | T | C | Yes |
| GATK.Unified.all | Full.Pipeline | NA18858 | 30432571 | T | C | T | C | Yes |
| GATK.Unified.all | Indel.Realignment | NA18858 | 30432571 | T | C | T | C | Yes |
| GATK.Unified.all | None | NA18858 | 30432571 | T | C | T | C | Yes |
| GATK.Unified.HQ | Base.Recalibration | NA18858 | 30432571 | T | C | T | C | Yes |
| GATK.Unified.HQ | Full.Pipeline | NA18858 | 30432571 | T | C | T | C | Yes |
| GATK.Unified.HQ | Indel.Realignment | NA18858 | 30432571 | T | C | T | C | Yes |
| GATK.Unified.HQ | None | NA18858 | 30432571 | T | C | T | C | Yes |
| VarScan | Base.Recalibration | NA18858 | 30432571 | T | C | T | C | Yes |
| VarScan | Full.Pipeline | NA18858 | 30432571 | T | C | T | C | Yes |
| VarScan | Indel.Realignment | NA18858 | 30432571 | T | C | T | C | Yes |
| VarScan | None | NA18858 | 30432571 | T | C | T | C | Yes |
| VarScan.custom | Base.Recalibration | NA18858 | 30432571 | T | C | T | C | Yes |
| VarScan.custom | Full.Pipeline | NA18858 | 30432571 | T | C | T | C | Yes |
| VarScan.custom | Indel.Realignment | NA18858 | 30432571 | T | C | T | C | Yes |
| VarScan.custom | None | NA18858 | 30432571 | T | C | T | C | Yes |
| VarScan.pvalue | Base.Recalibration | NA18858 | 30432571 | T | C | T | C | Yes |
| VarScan.pvalue | Full.Pipeline | NA18858 | 30432571 | T | C | T | C | Yes |
| VarScan.pvalue | Indel.Realignment | NA18858 | 30432571 | T | C | T | C | Yes |
| VarScan.pvalue | None | NA18858 | 30432571 | T | C | T | C | Yes |
| GATK.Haplotype.all | Base.Recalibration | NA18870 | 947854 | C | T | C | T | Yes |
| GATK.Haplotype.all | Full.Pipeline | NA18870 | 947854 | C | T | C | T | Yes |
| GATK.Haplotype.all | Indel.Realignment | NA18870 | 947854 | C | T | C | T | Yes |
| GATK.Haplotype.all | None | NA18870 | 947854 | C | T | C | T | Yes |
| GATK.Haplotype.HQ | Base.Recalibration | NA18870 | 947854 | C | T | C | T | Yes |
| GATK.Haplotype.HQ | Full.Pipeline | NA18870 | 947854 | C | T | C | T | Yes |
| GATK.Haplotype.HQ | Indel.Realignment | NA18870 | 947854 | C | T | C | T | Yes |
| GATK.Haplotype.HQ | None | NA18870 | 947854 | C | T | C | T | Yes |
| GATK.Unified.all | Base.Recalibration | NA18870 | 947854 | C | T | C | T | Yes |
| GATK.Unified.all | Full.Pipeline | NA18870 | 947854 | C | T | C | T | Yes |
| GATK.Unified.all | Indel.Realignment | NA18870 | 947854 | C | T | C | T | Yes |
| GATK.Unified.all | None | NA18870 | 947854 | C | T | C | T | Yes |
| GATK.Unified.HQ | Base.Recalibration | NA18870 | 947854 | C | T | C | T | Yes |
| GATK.Unified.HQ | Full.Pipeline | NA18870 | 947854 | C | T | C | T | Yes |
| GATK.Unified.HQ | Indel.Realignment | NA18870 | 947854 | C | T | C | T | Yes |
| GATK.Unified.HQ | None | NA18870 | 947854 | C | T | C | T | Yes |
| VarScan | Base.Recalibration | NA18870 | 947854 | C | T | C | T | Yes |
| VarScan | Full.Pipeline | NA18870 | 947854 | C | T | C | T | Yes |
| VarScan | Indel.Realignment | NA18870 | 947854 | C | T | C | T | Yes |
| VarScan | None | NA18870 | 947854 | C | T | C | T | Yes |
| VarScan.custom | Base.Recalibration | NA18870 | 947854 | C | T | C | T | Yes |
| VarScan.custom | Full.Pipeline | NA18870 | 947854 | C | T | C | T | Yes |
| VarScan.custom | Indel.Realignment | NA18870 | 947854 | C | T | C | T | Yes |
| VarScan.custom | None | NA18870 | 947854 | C | T | C | T | Yes |
| VarScan.pvalue | Base.Recalibration | NA18870 | 947854 | C | T | C | T | Yes |
| VarScan.pvalue | Full.Pipeline | NA18870 | 947854 | C | T | C | T | Yes |
| VarScan.pvalue | Indel.Realignment | NA18870 | 947854 | C | T | C | T | Yes |
| VarScan.pvalue | None | NA18870 | 947854 | C | T | C | T | Yes |
| GATK.Haplotype.all | Base.Recalibration | NA18870 | 17474782 | G | A | G | A | Yes |
| GATK.Haplotype.all | Full.Pipeline | NA18870 | 17474782 | G | A | G | A | Yes |
| GATK.Haplotype.all | Indel.Realignment | NA18870 | 17474782 | G | A | G | A | Yes |
| GATK.Haplotype.all | None | NA18870 | 17474782 | G | A | G | A | Yes |
| GATK.Haplotype.HQ | Base.Recalibration | NA18870 | 17474782 | G | A | G | A | Yes |
| GATK.Haplotype.HQ | Full.Pipeline | NA18870 | 17474782 | G | A | G | A | Yes |
| GATK.Haplotype.HQ | Indel.Realignment | NA18870 | 17474782 | G | A | G | A | Yes |
| GATK.Haplotype.HQ | None | NA18870 | 17474782 | G | A | G | A | Yes |
| GATK.Unified.all | Base.Recalibration | NA18870 | 17474782 | G | A | G | A | Yes |
| GATK.Unified.all | Full.Pipeline | NA18870 | 17474782 | G | A | G | A | Yes |
| GATK.Unified.all | Indel.Realignment | NA18870 | 17474782 | G | A | G | A | Yes |
| GATK.Unified.all | None | NA18870 | 17474782 | G | A | G | A | Yes |
| GATK.Unified.HQ | Base.Recalibration | NA18870 | 17474782 | G | A | G | A | Yes |
| GATK.Unified.HQ | Full.Pipeline | NA18870 | 17474782 | G | A | G | A | Yes |
| GATK.Unified.HQ | Indel.Realignment | NA18870 | 17474782 | G | A | G | A | Yes |
| GATK.Unified.HQ | None | NA18870 | 17474782 | G | A | G | A | Yes |
| VarScan | Base.Recalibration | NA18870 | 17474782 | G | A | G | A | Yes |
| VarScan | Full.Pipeline | NA18870 | 17474782 | G | A | G | A | Yes |
| VarScan | Indel.Realignment | NA18870 | 17474782 | G | A | G | A | Yes |
| VarScan | None | NA18870 | 17474782 | G | A | G | A | Yes |
| VarScan.custom | Base.Recalibration | NA18870 | 17474782 | G | A | G | A | Yes |
| VarScan.custom | Full.Pipeline | NA18870 | 17474782 | G | A | G | A | Yes |
| VarScan.custom | Indel.Realignment | NA18870 | 17474782 | G | A | G | A | Yes |
| VarScan.custom | None | NA18870 | 17474782 | G | A | G | A | Yes |
| VarScan.pvalue | Base.Recalibration | NA18870 | 17474782 | G | A | G | A | Yes |
| VarScan.pvalue | Full.Pipeline | NA18870 | 17474782 | G | A | G | A | Yes |
| VarScan.pvalue | Indel.Realignment | NA18870 | 17474782 | G | A | G | A | Yes |
| VarScan.pvalue | None | NA18870 | 17474782 | G | A | G | A | Yes |
| GATK.Haplotype.all | Base.Recalibration | NA18870 | 31021430 | G | C | G | C | Yes |
| GATK.Haplotype.all | Full.Pipeline | NA18870 | 31021430 | G | C | G | C | Yes |
| GATK.Haplotype.all | Indel.Realignment | NA18870 | 31021430 | G | C | G | C | Yes |
| GATK.Haplotype.all | None | NA18870 | 31021430 | G | C | G | C | Yes |
| GATK.Haplotype.HQ | Base.Recalibration | NA18870 | 31021430 | G | C | G | C | Yes |
| GATK.Haplotype.HQ | Full.Pipeline | NA18870 | 31021430 | G | C | G | C | Yes |
| GATK.Haplotype.HQ | Indel.Realignment | NA18870 | 31021430 | G | C | G | C | Yes |
| GATK.Haplotype.HQ | None | NA18870 | 31021430 | G | C | G | C | Yes |
| GATK.Unified.all | Base.Recalibration | NA18870 | 31021430 | G | C | G | C | Yes |
| GATK.Unified.all | Full.Pipeline | NA18870 | 31021430 | G | C | G | C | Yes |
| GATK.Unified.all | Indel.Realignment | NA18870 | 31021430 | G | C | G | C | Yes |
| GATK.Unified.all | None | NA18870 | 31021430 | G | C | G | C | Yes |
| GATK.Unified.HQ | Base.Recalibration | NA18870 | 31021430 | G | C | G | C | Yes |
| GATK.Unified.HQ | Full.Pipeline | NA18870 | 31021430 | G | C | G | C | Yes |
| GATK.Unified.HQ | Indel.Realignment | NA18870 | 31021430 | G | C | G | C | Yes |
| GATK.Unified.HQ | None | NA18870 | 31021430 | G | C | G | C | Yes |
| VarScan | Base.Recalibration | NA18870 | 31021430 | G | C | G | C | Yes |
| VarScan | Full.Pipeline | NA18870 | 31021430 | G | C | G | C | Yes |
| VarScan | Indel.Realignment | NA18870 | 31021430 | G | C | G | C | Yes |
| VarScan | None | NA18870 | 31021430 | G | C | G | C | Yes |
| VarScan.custom | Base.Recalibration | NA18870 | 31021430 | G | C | G | C | Yes |
| VarScan.custom | Full.Pipeline | NA18870 | 31021430 | G | C | G | C | Yes |
| VarScan.custom | Indel.Realignment | NA18870 | 31021430 | G | C | G | C | Yes |
| VarScan.custom | None | NA18870 | 31021430 | G | C | G | C | Yes |
| VarScan.pvalue | Base.Recalibration | NA18870 | 31021430 | G | C | G | C | Yes |
| VarScan.pvalue | Full.Pipeline | NA18870 | 31021430 | G | C | G | C | Yes |
| VarScan.pvalue | Indel.Realignment | NA18870 | 31021430 | G | C | G | C | Yes |
| VarScan.pvalue | None | NA18870 | 31021430 | G | C | G | C | Yes |
| GATK.Haplotype.all | Base.Recalibration | NA18870 | 31022765 | C | T | C | T | Yes |
| GATK.Haplotype.all | Full.Pipeline | NA18870 | 31022765 | C | T | C | T | Yes |
| GATK.Haplotype.all | Indel.Realignment | NA18870 | 31022765 | C | T | C | T | Yes |
| GATK.Haplotype.all | None | NA18870 | 31022765 | C | T | C | T | Yes |
| GATK.Haplotype.HQ | Base.Recalibration | NA18870 | 31022765 | C | T | C | T | Yes |
| GATK.Haplotype.HQ | Full.Pipeline | NA18870 | 31022765 | C | T | C | T | Yes |
| GATK.Haplotype.HQ | Indel.Realignment | NA18870 | 31022765 | C | T | C | T | Yes |
| GATK.Haplotype.HQ | None | NA18870 | 31022765 | C | T | C | T | Yes |
| GATK.Unified.all | Base.Recalibration | NA18870 | 31022765 | C | T | C | T | Yes |
| GATK.Unified.all | Full.Pipeline | NA18870 | 31022765 | C | T | C | T | Yes |
| GATK.Unified.all | Indel.Realignment | NA18870 | 31022765 | C | T | C | T | Yes |
| GATK.Unified.all | None | NA18870 | 31022765 | C | T | C | T | Yes |
| GATK.Unified.HQ | Base.Recalibration | NA18870 | 31022765 | C | T | C | T | Yes |
| GATK.Unified.HQ | Full.Pipeline | NA18870 | 31022765 | C | T | C | T | Yes |
| GATK.Unified.HQ | Indel.Realignment | NA18870 | 31022765 | C | T | C | T | Yes |
| GATK.Unified.HQ | None | NA18870 | 31022765 | C | T | C | T | Yes |
| VarScan | Base.Recalibration | NA18870 | 31022765 | C | T | C | T | Yes |
| VarScan | Full.Pipeline | NA18870 | 31022765 | C | T | C | T | Yes |
| VarScan | Indel.Realignment | NA18870 | 31022765 | C | T | C | T | Yes |
| VarScan | None | NA18870 | 31022765 | C | T | C | T | Yes |
| VarScan.custom | Base.Recalibration | NA18870 | 31022765 | C | T | C | T | Yes |
| VarScan.custom | Full.Pipeline | NA18870 | 31022765 | C | T | C | T | Yes |
| VarScan.custom | Indel.Realignment | NA18870 | 31022765 | C | T | C | T | Yes |
| VarScan.custom | None | NA18870 | 31022765 | C | T | C | T | Yes |
| VarScan.pvalue | Base.Recalibration | NA18870 | 31022765 | C | T | C | T | Yes |
| VarScan.pvalue | Full.Pipeline | NA18870 | 31022765 | C | T | C | T | Yes |
| VarScan.pvalue | Indel.Realignment | NA18870 | 31022765 | C | T | C | T | Yes |
| VarScan.pvalue | None | NA18870 | 31022765 | C | T | C | T | Yes |
| GATK.Haplotype.all | Base.Recalibration | NA18870 | 62326151 | G | A | G | A | Yes |
| GATK.Haplotype.all | Full.Pipeline | NA18870 | 62326151 | G | A | G | A | Yes |
| GATK.Haplotype.all | Indel.Realignment | NA18870 | 62326151 | G | A | G | A | Yes |
| GATK.Haplotype.all | None | NA18870 | 62326151 | G | A | G | A | Yes |
| GATK.Haplotype.HQ | Base.Recalibration | NA18870 | 62326151 | G | A | G | A | Yes |
| GATK.Haplotype.HQ | Full.Pipeline | NA18870 | 62326151 | G | A | G | A | Yes |
| GATK.Haplotype.HQ | Indel.Realignment | NA18870 | 62326151 | G | A | G | A | Yes |
| GATK.Haplotype.HQ | None | NA18870 | 62326151 | G | A | G | A | Yes |
| GATK.Unified.all | Base.Recalibration | NA18870 | 62326151 | G | A | G | A | Yes |
| GATK.Unified.all | Full.Pipeline | NA18870 | 62326151 | G | A | G | A | Yes |
| GATK.Unified.all | Indel.Realignment | NA18870 | 62326151 | G | A | G | A | Yes |
| GATK.Unified.all | None | NA18870 | 62326151 | G | A | G | A | Yes |
| GATK.Unified.HQ | Base.Recalibration | NA18870 | 62326151 | G | A | G | A | Yes |
| GATK.Unified.HQ | Full.Pipeline | NA18870 | 62326151 | G | A | G | A | Yes |
| GATK.Unified.HQ | Indel.Realignment | NA18870 | 62326151 | G | A | G | A | Yes |
| GATK.Unified.HQ | None | NA18870 | 62326151 | G | A | G | A | Yes |
| VarScan | Base.Recalibration | NA18870 | 62326151 | G | A | G | A | Yes |
| VarScan | Full.Pipeline | NA18870 | 62326151 | G | A | G | A | Yes |
| VarScan | Indel.Realignment | NA18870 | 62326151 | G | A | G | A | Yes |
| VarScan | None | NA18870 | 62326151 | G | A | G | A | Yes |
| VarScan.custom | Base.Recalibration | NA18870 | 62326151 | G | A | G | A | Yes |
| VarScan.custom | Full.Pipeline | NA18870 | 62326151 | G | A | G | A | Yes |
| VarScan.custom | Indel.Realignment | NA18870 | 62326151 | G | A | G | A | Yes |
| VarScan.custom | None | NA18870 | 62326151 | G | A | G | A | Yes |
| VarScan.pvalue | Base.Recalibration | NA18870 | 62326151 | G | A | G | A | Yes |
| VarScan.pvalue | Full.Pipeline | NA18870 | 62326151 | G | A | G | A | Yes |
| VarScan.pvalue | Indel.Realignment | NA18870 | 62326151 | G | A | G | A | Yes |
| VarScan.pvalue | None | NA18870 | 62326151 | G | A | G | A | Yes |
| GATK.Haplotype.all | Base.Recalibration | NA19000 | 20020490 | T | C | T | C | Yes |
| GATK.Haplotype.all | Full.Pipeline | NA19000 | 20020490 | T | C | T | C | Yes |
| GATK.Haplotype.all | Indel.Realignment | NA19000 | 20020490 | T | C | T | C | Yes |
| GATK.Haplotype.all | None | NA19000 | 20020490 | T | C | T | C | Yes |
| GATK.Haplotype.HQ | Base.Recalibration | NA19000 | 20020490 | T | C | T | C | Yes |
| GATK.Haplotype.HQ | Full.Pipeline | NA19000 | 20020490 | T | C | T | C | Yes |
| GATK.Haplotype.HQ | Indel.Realignment | NA19000 | 20020490 | T | C | T | C | Yes |
| GATK.Haplotype.HQ | None | NA19000 | 20020490 | T | C | T | C | Yes |
| GATK.Unified.all | Base.Recalibration | NA19000 | 20020490 | T | C | T | C | Yes |
| GATK.Unified.all | Full.Pipeline | NA19000 | 20020490 | T | C | T | C | Yes |
| GATK.Unified.all | Indel.Realignment | NA19000 | 20020490 | T | C | T | C | Yes |
| GATK.Unified.all | None | NA19000 | 20020490 | T | C | T | C | Yes |
| GATK.Unified.HQ | Base.Recalibration | NA19000 | 20020490 | T | C | T | C | Yes |
| GATK.Unified.HQ | Full.Pipeline | NA19000 | 20020490 | T | C | T | C | Yes |
| GATK.Unified.HQ | Indel.Realignment | NA19000 | 20020490 | T | C | T | C | Yes |
| GATK.Unified.HQ | None | NA19000 | 20020490 | T | C | T | C | Yes |
| VarScan | Base.Recalibration | NA19000 | 20020490 | T | C | T | C | Yes |
| VarScan | Full.Pipeline | NA19000 | 20020490 | T | C | T | C | Yes |
| VarScan | Indel.Realignment | NA19000 | 20020490 | T | C | T | C | Yes |
| VarScan | None | NA19000 | 20020490 | T | C | T | C | Yes |
| VarScan.custom | Base.Recalibration | NA19000 | 20020490 | T | C | T | C | Yes |
| VarScan.custom | Full.Pipeline | NA19000 | 20020490 | T | C | T | C | Yes |
| VarScan.custom | Indel.Realignment | NA19000 | 20020490 | T | C | T | C | Yes |
| VarScan.custom | None | NA19000 | 20020490 | T | C | T | C | Yes |
| VarScan.pvalue | Base.Recalibration | NA19000 | 20020490 | T | C | T | C | Yes |
| VarScan.pvalue | Full.Pipeline | NA19000 | 20020490 | T | C | T | C | Yes |
| VarScan.pvalue | Indel.Realignment | NA19000 | 20020490 | T | C | T | C | Yes |
| VarScan.pvalue | None | NA19000 | 20020490 | T | C | T | C | Yes |
| GATK.Haplotype.all | Base.Recalibration | NA19000 | 23965922 | G | A | NA | NA | **No** |
| GATK.Haplotype.all | Full.Pipeline | NA19000 | 23965922 | G | A | NA | NA | **No** |
| GATK.Haplotype.all | Indel.Realignment | NA19000 | 23965922 | G | A | NA | NA | **No** |
| GATK.Haplotype.all | None | NA19000 | 23965922 | G | A | NA | NA | **No** |
| GATK.Haplotype.HQ | Base.Recalibration | NA19000 | 23965922 | G | A | NA | NA | **No** |
| GATK.Haplotype.HQ | Full.Pipeline | NA19000 | 23965922 | G | A | NA | NA | **No** |
| GATK.Haplotype.HQ | Indel.Realignment | NA19000 | 23965922 | G | A | NA | NA | **No** |
| GATK.Haplotype.HQ | None | NA19000 | 23965922 | G | A | NA | NA | **No** |
| GATK.Unified.all | Base.Recalibration | NA19000 | 23965922 | G | A | NA | NA | **No** |
| GATK.Unified.all | Full.Pipeline | NA19000 | 23965922 | G | A | NA | NA | **No** |
| GATK.Unified.all | Indel.Realignment | NA19000 | 23965922 | G | A | NA | NA | **No** |
| GATK.Unified.all | None | NA19000 | 23965922 | G | A | NA | NA | **No** |
| GATK.Unified.HQ | Base.Recalibration | NA19000 | 23965922 | G | A | NA | NA | **No** |
| GATK.Unified.HQ | Full.Pipeline | NA19000 | 23965922 | G | A | NA | NA | **No** |
| GATK.Unified.HQ | Indel.Realignment | NA19000 | 23965922 | G | A | NA | NA | **No** |
| GATK.Unified.HQ | None | NA19000 | 23965922 | G | A | NA | NA | **No** |
| VarScan | Base.Recalibration | NA19000 | 23965922 | G | A | G | A | Yes |
| VarScan | Full.Pipeline | NA19000 | 23965922 | G | A | G | A | Yes |
| VarScan | Indel.Realignment | NA19000 | 23965922 | G | A | G | A | Yes |
| VarScan | None | NA19000 | 23965922 | G | A | G | A | Yes |
| VarScan.custom | Base.Recalibration | NA19000 | 23965922 | G | A | NA | NA | **No** |
| VarScan.custom | Full.Pipeline | NA19000 | 23965922 | G | A | NA | NA | **No** |
| VarScan.custom | Indel.Realignment | NA19000 | 23965922 | G | A | NA | NA | **No** |
| VarScan.custom | None | NA19000 | 23965922 | G | A | NA | NA | **No** |
| VarScan.pvalue | Base.Recalibration | NA19000 | 23965922 | G | A | NA | NA | **No** |
| VarScan.pvalue | Full.Pipeline | NA19000 | 23965922 | G | A | NA | NA | **No** |
| VarScan.pvalue | Indel.Realignment | NA19000 | 23965922 | G | A | NA | NA | **No** |
| VarScan.pvalue | None | NA19000 | 23965922 | G | A | NA | NA | **No** |
| GATK.Haplotype.all | Base.Recalibration | NA19058 | 18440905 | G | C | G | C | Yes |
| GATK.Haplotype.all | Full.Pipeline | NA19058 | 18440905 | G | C | G | C | Yes |
| GATK.Haplotype.all | Indel.Realignment | NA19058 | 18440905 | G | C | G | C | Yes |
| GATK.Haplotype.all | None | NA19058 | 18440905 | G | C | G | C | Yes |
| GATK.Haplotype.HQ | Base.Recalibration | NA19058 | 18440905 | G | C | G | C | Yes |
| GATK.Haplotype.HQ | Full.Pipeline | NA19058 | 18440905 | G | C | G | C | Yes |
| GATK.Haplotype.HQ | Indel.Realignment | NA19058 | 18440905 | G | C | G | C | Yes |
| GATK.Haplotype.HQ | None | NA19058 | 18440905 | G | C | G | C | Yes |
| GATK.Unified.all | Base.Recalibration | NA19058 | 18440905 | G | C | G | C | Yes |
| GATK.Unified.all | Full.Pipeline | NA19058 | 18440905 | G | C | G | C | Yes |
| GATK.Unified.all | Indel.Realignment | NA19058 | 18440905 | G | C | G | C | Yes |
| GATK.Unified.all | None | NA19058 | 18440905 | G | C | G | C | Yes |
| GATK.Unified.HQ | Base.Recalibration | NA19058 | 18440905 | G | C | G | C | Yes |
| GATK.Unified.HQ | Full.Pipeline | NA19058 | 18440905 | G | C | G | C | Yes |
| GATK.Unified.HQ | Indel.Realignment | NA19058 | 18440905 | G | C | G | C | Yes |
| GATK.Unified.HQ | None | NA19058 | 18440905 | G | C | G | C | Yes |
| VarScan | Base.Recalibration | NA19058 | 18440905 | G | C | G | C | Yes |
| VarScan | Full.Pipeline | NA19058 | 18440905 | G | C | G | C | Yes |
| VarScan | Indel.Realignment | NA19058 | 18440905 | G | C | G | C | Yes |
| VarScan | None | NA19058 | 18440905 | G | C | G | C | Yes |
| VarScan.custom | Base.Recalibration | NA19058 | 18440905 | G | C | G | C | Yes |
| VarScan.custom | Full.Pipeline | NA19058 | 18440905 | G | C | G | C | Yes |
| VarScan.custom | Indel.Realignment | NA19058 | 18440905 | G | C | G | C | Yes |
| VarScan.custom | None | NA19058 | 18440905 | G | C | G | C | Yes |
| VarScan.pvalue | Base.Recalibration | NA19058 | 18440905 | G | C | G | C | Yes |
| VarScan.pvalue | Full.Pipeline | NA19058 | 18440905 | G | C | G | C | Yes |
| VarScan.pvalue | Indel.Realignment | NA19058 | 18440905 | G | C | G | C | Yes |
| VarScan.pvalue | None | NA19058 | 18440905 | G | C | G | C | Yes |
| GATK.Haplotype.all | Base.Recalibration | NA19058 | 42333998 | C | T | C | T | Yes |
| GATK.Haplotype.all | Full.Pipeline | NA19058 | 42333998 | C | T | C | T | Yes |
| GATK.Haplotype.all | Indel.Realignment | NA19058 | 42333998 | C | T | C | T | Yes |
| GATK.Haplotype.all | None | NA19058 | 42333998 | C | T | C | T | Yes |
| GATK.Haplotype.HQ | Base.Recalibration | NA19058 | 42333998 | C | T | C | T | Yes |
| GATK.Haplotype.HQ | Full.Pipeline | NA19058 | 42333998 | C | T | C | T | Yes |
| GATK.Haplotype.HQ | Indel.Realignment | NA19058 | 42333998 | C | T | C | T | Yes |
| GATK.Haplotype.HQ | None | NA19058 | 42333998 | C | T | C | T | Yes |
| GATK.Unified.all | Base.Recalibration | NA19058 | 42333998 | C | T | C | T | Yes |
| GATK.Unified.all | Full.Pipeline | NA19058 | 42333998 | C | T | C | T | Yes |
| GATK.Unified.all | Indel.Realignment | NA19058 | 42333998 | C | T | C | T | Yes |
| GATK.Unified.all | None | NA19058 | 42333998 | C | T | C | T | Yes |
| GATK.Unified.HQ | Base.Recalibration | NA19058 | 42333998 | C | T | C | T | Yes |
| GATK.Unified.HQ | Full.Pipeline | NA19058 | 42333998 | C | T | C | T | Yes |
| GATK.Unified.HQ | Indel.Realignment | NA19058 | 42333998 | C | T | C | T | Yes |
| GATK.Unified.HQ | None | NA19058 | 42333998 | C | T | C | T | Yes |
| VarScan | Base.Recalibration | NA19058 | 42333998 | C | T | C | T | Yes |
| VarScan | Full.Pipeline | NA19058 | 42333998 | C | T | C | T | Yes |
| VarScan | Indel.Realignment | NA19058 | 42333998 | C | T | C | T | Yes |
| VarScan | None | NA19058 | 42333998 | C | T | C | T | Yes |
| VarScan.custom | Base.Recalibration | NA19058 | 42333998 | C | T | C | T | Yes |
| VarScan.custom | Full.Pipeline | NA19058 | 42333998 | C | T | C | T | Yes |
| VarScan.custom | Indel.Realignment | NA19058 | 42333998 | C | T | C | T | Yes |
| VarScan.custom | None | NA19058 | 42333998 | C | T | C | T | Yes |
| VarScan.pvalue | Base.Recalibration | NA19058 | 42333998 | C | T | C | T | Yes |
| VarScan.pvalue | Full.Pipeline | NA19058 | 42333998 | C | T | C | T | Yes |
| VarScan.pvalue | Indel.Realignment | NA19058 | 42333998 | C | T | C | T | Yes |
| VarScan.pvalue | None | NA19058 | 42333998 | C | T | C | T | Yes |
| GATK.Haplotype.all | Base.Recalibration | NA19087 | 16253908 | C | A | C | A | Yes |
| GATK.Haplotype.all | Full.Pipeline | NA19087 | 16253908 | C | A | C | A | Yes |
| GATK.Haplotype.all | Indel.Realignment | NA19087 | 16253908 | C | A | C | A | Yes |
| GATK.Haplotype.all | None | NA19087 | 16253908 | C | A | C | A | Yes |
| GATK.Haplotype.HQ | Base.Recalibration | NA19087 | 16253908 | C | A | C | A | Yes |
| GATK.Haplotype.HQ | Full.Pipeline | NA19087 | 16253908 | C | A | C | A | Yes |
| GATK.Haplotype.HQ | Indel.Realignment | NA19087 | 16253908 | C | A | C | A | Yes |
| GATK.Haplotype.HQ | None | NA19087 | 16253908 | C | A | C | A | Yes |
| GATK.Unified.all | Base.Recalibration | NA19087 | 16253908 | C | A | C | A | Yes |
| GATK.Unified.all | Full.Pipeline | NA19087 | 16253908 | C | A | C | A | Yes |
| GATK.Unified.all | Indel.Realignment | NA19087 | 16253908 | C | A | C | A | Yes |
| GATK.Unified.all | None | NA19087 | 16253908 | C | A | C | A | Yes |
| GATK.Unified.HQ | Base.Recalibration | NA19087 | 16253908 | C | A | C | A | Yes |
| GATK.Unified.HQ | Full.Pipeline | NA19087 | 16253908 | C | A | C | A | Yes |
| GATK.Unified.HQ | Indel.Realignment | NA19087 | 16253908 | C | A | C | A | Yes |
| GATK.Unified.HQ | None | NA19087 | 16253908 | C | A | C | A | Yes |
| VarScan | Base.Recalibration | NA19087 | 16253908 | C | A | C | A | Yes |
| VarScan | Full.Pipeline | NA19087 | 16253908 | C | A | C | A | Yes |
| VarScan | Indel.Realignment | NA19087 | 16253908 | C | A | C | A | Yes |
| VarScan | None | NA19087 | 16253908 | C | A | C | A | Yes |
| VarScan.custom | Base.Recalibration | NA19087 | 16253908 | C | A | C | A | Yes |
| VarScan.custom | Full.Pipeline | NA19087 | 16253908 | C | A | C | A | Yes |
| VarScan.custom | Indel.Realignment | NA19087 | 16253908 | C | A | C | A | Yes |
| VarScan.custom | None | NA19087 | 16253908 | C | A | C | A | Yes |
| VarScan.pvalue | Base.Recalibration | NA19087 | 16253908 | C | A | C | A | Yes |
| VarScan.pvalue | Full.Pipeline | NA19087 | 16253908 | C | A | C | A | Yes |
| VarScan.pvalue | Indel.Realignment | NA19087 | 16253908 | C | A | C | A | Yes |
| VarScan.pvalue | None | NA19087 | 16253908 | C | A | C | A | Yes |
| GATK.Haplotype.all | Base.Recalibration | NA19087 | 55021022 | G | A | G | A | Yes |
| GATK.Haplotype.all | Full.Pipeline | NA19087 | 55021022 | G | A | G | A | Yes |
| GATK.Haplotype.all | Indel.Realignment | NA19087 | 55021022 | G | A | G | A | Yes |
| GATK.Haplotype.all | None | NA19087 | 55021022 | G | A | G | A | Yes |
| GATK.Haplotype.HQ | Base.Recalibration | NA19087 | 55021022 | G | A | G | A | Yes |
| GATK.Haplotype.HQ | Full.Pipeline | NA19087 | 55021022 | G | A | G | A | Yes |
| GATK.Haplotype.HQ | Indel.Realignment | NA19087 | 55021022 | G | A | G | A | Yes |
| GATK.Haplotype.HQ | None | NA19087 | 55021022 | G | A | G | A | Yes |
| GATK.Unified.all | Base.Recalibration | NA19087 | 55021022 | G | A | G | A | Yes |
| GATK.Unified.all | Full.Pipeline | NA19087 | 55021022 | G | A | G | A | Yes |
| GATK.Unified.all | Indel.Realignment | NA19087 | 55021022 | G | A | G | A | Yes |
| GATK.Unified.all | None | NA19087 | 55021022 | G | A | G | A | Yes |
| GATK.Unified.HQ | Base.Recalibration | NA19087 | 55021022 | G | A | G | A | Yes |
| GATK.Unified.HQ | Full.Pipeline | NA19087 | 55021022 | G | A | G | A | Yes |
| GATK.Unified.HQ | Indel.Realignment | NA19087 | 55021022 | G | A | G | A | Yes |
| GATK.Unified.HQ | None | NA19087 | 55021022 | G | A | G | A | Yes |
| VarScan | Base.Recalibration | NA19087 | 55021022 | G | A | G | A | Yes |
| VarScan | Full.Pipeline | NA19087 | 55021022 | G | A | G | A | Yes |
| VarScan | Indel.Realignment | NA19087 | 55021022 | G | A | G | A | Yes |
| VarScan | None | NA19087 | 55021022 | G | A | G | A | Yes |
| VarScan.custom | Base.Recalibration | NA19087 | 55021022 | G | A | G | A | Yes |
| VarScan.custom | Full.Pipeline | NA19087 | 55021022 | G | A | G | A | Yes |
| VarScan.custom | Indel.Realignment | NA19087 | 55021022 | G | A | G | A | Yes |
| VarScan.custom | None | NA19087 | 55021022 | G | A | G | A | Yes |
| VarScan.pvalue | Base.Recalibration | NA19087 | 55021022 | G | A | G | A | Yes |
| VarScan.pvalue | Full.Pipeline | NA19087 | 55021022 | G | A | G | A | Yes |
| VarScan.pvalue | Indel.Realignment | NA19087 | 55021022 | G | A | G | A | Yes |
| VarScan.pvalue | None | NA19087 | 55021022 | G | A | G | A | Yes |
