# Supplementary material for: Detailed comparison of two popular variant calling packages for exome and targeted exon studies
Source: PeerJ. 2014 Sep 30;2:e600. doi: 10.7717/peerj.600 (PMC4184249; doi:10.7717/peerj.600)
Supplement: Table S7 [file peerj-02-600-s026.doc]

**Table S7: Recovery of Human610 SNP Chip Variants for SRP19719 Subject K8108-49685s**

| **Variant.Caller** | **Preprocessing** | **Total.Variants** | **Validated.Variants** | **Concordance.Rate** |
| --- | --- | --- | --- | --- |
| GATK HaplotypeCaller  (all) | No Preprocessing | 6437 | 6144 | 95.44819 |
|  | Realign Only | 6437 | 6144 | 95.44819 |
|  | Recalibrate Only | 6437 | 6145 | 95.46373 |
|  | Full Pipeline | 6437 | 6146 | 95.47926 |
| GATK HaplotypeCaller  (High-Quality) | No Preprocessing | 6437 | 6119 | 95.05981 |
|  | Realign Only | 6437 | 6119 | 95.05981 |
|  | Recalibrate Only | 6437 | 6122 | 95.10642 |
|  | Full Pipeline | 6437 | 6122 | 95.10642 |
| GATK Unified Genotyper  (all) | No Preprocessing | 6437 | 6176 | 95.94532 |
|  | Realign Only | 6437 | 6176 | 95.94532 |
|  | Recalibrate Only | 6437 | 6173 | 95.89871 |
|  | Full Pipeline | 6437 | 6173 | 95.89871 |
| GATK Unified Genotyper  (High-Quality) | No Preprocessing | 6437 | 6131 | 95.24623 |
|  | Realign Only | 6437 | 6131 | 95.24623 |
|  | Recalibrate Only | 6437 | 6126 | 95.16856 |
|  | Full Pipeline | 6437 | 6126 | 95.16856 |
| VarScan  (Default) | No Preprocessing | 6437 | 5912 | 91.84403 |
|  | Realign Only | 6437 | 5912 | 91.84403 |
|  | Recalibrate Only | 6437 | 5923 | 92.01491 |
|  | Full Pipeline | 6437 | 5923 | 92.01491 |
| VarScan  (Custom) | No Preprocessing | 6437 | 5684 | 88.302 |
|  | Realign Only | 6437 | 5684 | 88.302 |
|  | Recalibrate Only | 6437 | 5693 | 88.44182 |
|  | Full Pipeline | 6437 | 5693 | 88.44182 |
| VarScan  (P-value) | No Preprocessing | 6437 | 5856 | 90.97406 |
|  | Realign Only | 6437 | 5856 | 90.97406 |
|  | Recalibrate Only | 6437 | 5866 | 91.12941 |
|  | Full Pipeline | 6437 | 5866 | 91.12941 |
