# Supplementary material for: Detailed comparison of two popular variant calling packages for exome and targeted exon studies
Source: PeerJ. 2014 Sep 30;2:e600. doi: 10.7717/peerj.600 (PMC4184249; doi:10.7717/peerj.600)
Supplement: Table S8 — Validation Rates Confined to Targeted Exon Regions on chromosome 20. There were no unknown frequency variants called within these regions for VarScan-Cons. “HQ” = “High-Quality” [file peerj-02-600-s027.doc]

**Table S8: Validation Rate for Unknown Frequency SNPs**

| **Variant Caller** | **Preprocessing** | **Total Variants** | **Validated Variants** | **Concordance Rate** |
| --- | --- | --- | --- | --- |
| GATK.Haplotype.all | Base.Recalibration | 4 | 1 | 25% |
|  | Full.Pipeline | 4 | 1 | 25% |
|  | Indel.Realignment | 4 | 1 | 25% |
|  | None | 4 | 1 | 25% |
| GATK.Haplotype.HQ | Base.Recalibration | 2 | 1 | 50% |
|  | Full.Pipeline | 2 | 1 | 50% |
|  | Indel.Realignment | 3 | 1 | 33% |
|  | None | 3 | 1 | 33% |
| GATK.Unified.all | Base.Recalibration | 10 | 2 | 20% |
|  | Full.Pipeline | 10 | 1 | 10% |
|  | Indel.Realignment | 22 | 1 | 5% |
|  | None | 11 | 1 | 9% |
| GATK.Unified.HQ | Base.Recalibration | 5 | 1 | 20% |
|  | Full.Pipeline | 5 | 1 | 20% |
|  | Indel.Realignment | 11 | 1 | 9% |
|  | None | 11 | 1 | 9% |
| VarScan | Base.Recalibration | 2344 | 19 | **<1%** |
|  | Full.Pipeline | 2343 | 19 | **<1%** |
|  | Indel.Realignment | 3634 | 32 | **<1%** |
|  | None | 3634 | 32 | **<1%** |
| VarScan.pvalue | Base.Recalibration | 45 | 1 | 2% |
|  | Full.Pipeline | 45 | 1 | 2% |
|  | Indel.Realignment | 201 | 1 | **<1%** |
|  | None | 201 | 1 | **<1%** |

Validation Rates Confined to Targeted Exon Regions on chromosome 20. There were no unknown frequency variants called within these regions for VarScan-Cons. “HQ” = “High-Quality”
