# Supplementary material for: Detailed comparison of two popular variant calling packages for exome and targeted exon studies
Source: PeerJ. 2014 Sep 30;2:e600. doi: 10.7717/peerj.600 (PMC4184249; doi:10.7717/peerj.600)
Supplement: Table S9 [file peerj-02-600-s028.doc]

**Table S9: Variant Counts for SRP Amplicon Sample (SRR850313)**

1. **SNP Counts**

| **Variant.Caller** | **Caller.Details** | **Num.SNP.no.preprocess** | **Num.SNP.realign** | **Num.SNP.recal** | **Num.SNP.full.pipeline** |
| --- | --- | --- | --- | --- | --- |
| VarScan | Default | 16,672 | 16,591 | 17,713 | 17,644 |
| VarScan | Pvalue | 11,709 | 11,645 | 11,853 | 11,796 |
| VarScan | Custom | 3,326 | 3,300 | 4,016 | 3,983 |
| GATK | Unified.all | 16,929 | 16,796 | 13,336 | 13,274 |
| GATK | Unified.highQuality | 12,726 | 12,592 | 11,363 | 11,304 |
| GATK | Haplotype.all | 5,922 | 5,906 | 5,410 | 5,448 |
| GATK | Haplotype.highQuality | 5,436 | 5,416 | 5,090 | 5,118 |

1. **Indel Counts**

| **Variant.Caller** | **Caller.Details** | **Num.Indel.no.preprocess** | **Num.Indel.realign** | **Num.Indel.recal** | **Num.Indel.full.pipeline** |
| --- | --- | --- | --- | --- | --- |
| VarScan | Default | 2,597 | 2,618 | 2,600 | 2,613 |
| VarScan | Pvalue | 2,058 | 2,060 | 2,057 | 2,053 |
| VarScan | Custom | 797 | 811 | 801 | 808 |
| GATK | Unified.all | 839 | 872 | 834 | 860 |
| GATK | Unified.highQuality | 837 | 867 | 829 | 857 |
| GATK | Haplotype.all | 1636 | 1632 | 1551 | 1552 |
| GATK | Haplotype.highQuality | 1585 | 1581 | 1521 | 1523 |
