# Supplementary material for: Detailed comparison of two popular variant calling packages for exome and targeted exon studies
Source: PeerJ. 2014 Sep 30;2:e600. doi: 10.7717/peerj.600 (PMC4184249; doi:10.7717/peerj.600)
Supplement: Table S10 [file peerj-02-600-s029.doc]

**Table S10: Novel Variant Frequencies for SRP Amplicon Sample (SRR850313)**

| **Variant Caller** | **Preprocessing** | **Novel Variant Percentage** |
| --- | --- | --- |
| GATK HaplotypeCaller  (all) | None | 33.1% |
|  | Realign Only | 32.1% |
|  | Recalibrate Only | 30.8% |
|  | Full Pipeline | 31.1% |
| GATK HaplotypeCaller  (High-Quality) | None | 31.5% |
|  | Realign Only | 30.5% |
|  | Recalibrate Only | 29.8% |
|  | Full Pipeline | 29.8% |
| GATK Unified Genotyper  (all) | None | 73.1% |
|  | Realign Only | 72.9% |
|  | Recalibrate Only | 69.6% |
|  | Full Pipeline | 69.7% |
| GATK Unified Genotyper  (High-Quality) | None | 68.9% |
|  | Realign Only | 68.5% |
|  | Recalibrate Only | 66.3% |
|  | Full Pipeline | 66.1% |
| VarScan  (Default) | None | 66.7% |
|  | Realign Only | 66.6% |
|  | Recalibrate Only | 67.6% |
|  | Full Pipeline | 67.6% |
| VarScan  (P-value) | None | 60.1% |
|  | Realign Only | 59.9% |
|  | Recalibrate Only | 59.1% |
|  | Full Pipeline | 59.0% |
| VarScan  (Custom) | None | 30.0% |
|  | Realign Only | 29.4% |
|  | Recalibrate Only | 33.6% |
|  | Full Pipeline | 33.0% |
