# Supplementary material for: Detailed comparison of two popular variant calling packages for exome and targeted exon studies
Source: PeerJ. 2014 Sep 30;2:e600. doi: 10.7717/peerj.600 (PMC4184249; doi:10.7717/peerj.600)
Supplement: Table S11 — Validation Rates Confined to Targeted Exon Regions on chromosome 20. Variants are all subset of variants called with VarScan using default parameters and all possible pre-processing steps. [file peerj-02-600-s030.doc]

**Table S11: Impact of Supporting Read Frequency on 1KG Targeted Exon Variant Recovery**

|  |  | **Previously Observed** | | | **Novel** | | |
| --- | --- | --- | --- | --- | --- | --- | --- |
| **Variant Read Percentage**  **(Minimum)** | **Number of Variant Reads**  **(Minimum)** | **Total Variants** | **Validated Variants** | **Concordance Rate** | **Total Variants** | **Validated Variants** | **Concordance Rate** |
| 1% | 2 | 309 | 244 | **78.9644** | 2343 | 19 | **0.810926** |
|  | 4 | 246 | 231 | **93.90244** | 92 | 2 | **2.173913** |
|  | 10 | 199 | 181 | **90.95477** | 7 | 1 | **14.28571** |
|  | 20 | 134 | 117 | **87.31343** | 1 | 0 | **0** |
| 5% | 2 | 263 | 239 | **90.87452** | 219 | 4 | **1.826484** |
|  | 4 | 245 | 229 | **93.46939** | 35 | 2 | **5.714286** |
|  | 10 | 199 | 181 | **90.95477** | 7 | 1 | **14.28571** |
|  | 20 | 134 | 117 | **87.31343** | 1 | 0 | **0** |
| 15% | 2 | 245 | 232 | **94.69388** | 22 | 1 | **4.545455** |
|  | 4 | 235 | 226 | **96.17021** | 8 | 1 | **12.5** |
|  | 10 | 197 | 180 | **91.37056** | 4 | 1 | **25** |
|  | 20 | 134 | 117 | **87.31343** | 1 | 0 | **0** |
| 30% | 2 | 221 | 214 | 96.83258 | 0 | 0 | NA |
|  | 4 | 219 | 213 | 97.26027 | 0 | 0 | NA |
|  | 10 | 186 | 175 | 94.08602 | 0 | 0 | NA |
|  | 20 | 126 | 114 | 90.47619 | 0 | 0 | NA |
| 50% | 2 | 108 | 81 | 75 | 0 | 0 | NA |
|  | 4 | 107 | 81 | 75.70093 | 0 | 0 | NA |
|  | 10 | 96 | 73 | 76.04167 | 0 | 0 | NA |
|  | 20 | 62 | 49 | 79.03226 | 0 | 0 | NA |
| 90% | 2 | 66 | 65 | 98.48485 | 0 | 0 | NA |
|  | 4 | 65 | 65 | 100 | 0 | 0 | NA |
|  | 10 | 60 | 60 | 100 | 0 | 0 | NA |
|  | 20 | 43 | 42 | 97.67442 | 0 | 0 | NA |

Validation Rates Confined to Targeted Exon Regions on chromosome 20. Variants are all subset of variants called with VarScan using default parameters and all possible pre-processing steps.
