# Supplementary material for: Detailed comparison of two popular variant calling packages for exome and targeted exon studies
Source: PeerJ. 2014 Sep 30;2:e600. doi: 10.7717/peerj.600 (PMC4184249; doi:10.7717/peerj.600)
Supplement: Table S12 — Replicates for same individual highlighted in green and yellow [file peerj-02-600-s031.doc]

**Table S12: Run-Times for 1KG Targeted Exon Samples**

1. Run-times for individual steps

| **Pipeline** | **Preprocessing** | **ERR002985** | **ERR002993** | **ERR002994** | **ERR003014** | **ERR003016** | **ERR004078** | **ERR004084** | **SRR013632** | **SRR013635** | **SRR013654** | **SRR013709** | **SRR017908** | **SRR017914** | **SRR018122** |
| --- | --- | --- | --- | --- | --- | --- | --- | --- | --- | --- | --- | --- | --- | --- | --- |
| Both | Filter singletons (GATK) | 0:01 | 0:02 | 0:02 | 0:04 | 0:03 | 0:08 | 0:04 | 0:04 | 0:05 | 0:05 | 0:04 | 0:06 | 0:02 | 0:07 |
| Both | sort sample | 0:02 | 0:02 | 0:01 | 0:06 | 0:04 | 0:09 | 0:06 | 0:04 | 0:05 | 0:05 | 0:05 | 0:08 | 0:04 | 0:08 |
| Both | remove duplicates | 0:03 | 0:04 | 0:03 | 0:08 | 0:06 | 0:11 | 0:08 | 0:05 | 0:07 | 0:06 | 0:05 | 0:10 | 0:03 | 0:10 |
| GATK | add read groups + karyotype reorder (GATK) | 0:03 | 0:03 | 0:03 | 0:07 | 0:05 | 0:07 | 0:10 | 0:03 | 0:03 | 0:04 | 0:04 | 0:09 | 0:01 | 0:07 |
| VarScan | pileup + clean pileup (VarScan, no preprocess) | 0:13 | 0:10 | 0:10 | 0:23 | 0:21 | 0:26 | 0:22 | 0:27 | 0:14 | 0:17 | 0:19 | 0:41 | 0:05 | 0:30 |
| VarScan | varscan default (no preprocess) | 0:06 | 0:07 | 0:06 | 0:11 | 0:11 | 0:13 | 0:12 | 0:05 | 0:06 | 0:08 | 0:10 | 0:20 | 0:03 | 0:10 |
| VarScan | varscan pvalue (no preprocess) | 0:07 | 0:06 | 0:05 | 0:12 | 0:10 | 0:14 | 0:12 | 0:04 | 0:06 | 0:08 | 0:10 | 0:19 | 0:03 | 0:10 |
| VarScan | varscan custom (no preprocess) | 0:07 | 0:06 | 0:05 | 0:11 | 0:10 | 0:13 | 0:12 | 0:05 | 0:05 | 0:08 | 0:09 | 0:19 | 0:03 | 0:09 |
| GATK | GATK Haplotype (no preprocess) | 0:55 | 0:45 | 0:43 | 1:19 | 2:04 | 1:29 | 2:23 | 2:09 | 1:08 | 1:51 | 1:30 | 3:07 | 0:41 | 1:52 |
| GATK | GATK Unified (no preprocess) | 1:45 | 1:38 | 1:43 | 2:58 | 1:51 | 3:01 | 1:53 | 1:53 | 1:54 | 2:54 | 1:50 | 2:21 | 1:33 | 2:04 |
| Both | GATK realign | 0:21 | 0:20 | 0:20 | 0:25 | 0:23 | 0:27 | 0:26 | 0:23 | 0:21 | 0:22 | 0:23 | 0:33 | 0:16 | 0:28 |
| VarScan | pileup + clean pileup (VarScan, realign) | 0:12 | 0:10 | 0:11 | 0:22 | 0:20 | 0:26 | 0:22 | 0:17 | 0:14 | 0:17 | 0:19 | 0:40 | 0:05 | 0:30 |
| VarScan | varscan default (realign) | 0:07 | 0:07 | 0:05 | 0:12 | 0:11 | 0:13 | 0:12 | 0:04 | 0:06 | 0:09 | 0:09 | 0:20 | 0:03 | 0:09 |
| VarScan | varscan pvalue (realign) | 0:07 | 0:06 | 0:06 | 0:11 | 0:11 | 0:14 | 0:12 | 0:05 | 0:06 | 0:08 | 0:10 | 0:20 | 0:03 | 0:10 |
| VarScan | varscan custom (realign) | 0:07 | 0:07 | 0:05 | 0:12 | 0:10 | 0:13 | 0:11 | 0:04 | 0:06 | 0:08 | 0:10 | 0:20 | 0:03 | 0:09 |
| GATK | GATK Haplotype (realign) | 0:59 | 0:45 | 0:46 | 1:18 | 1:08 | 1:29 | 1:21 | 1:08 | 1:06 | 2:02 | 1:27 | 3:10 | 0:40 | 1:51 |
| GATK | GATK Unified (realign) | 1:44 | 1:40 | 1:54 | 1:51 | 1:51 | 1:59 | 1:52 | 1:41 | 1:48 | 1:59 | 1:44 | 2:26 | 1:34 | 1:56 |
| Both | GATK recal (w/o realign) | 0:21 | 0:21 | 0:30 | 0:42 | 0:31 | 0:40 | 0:43 | 0:34 | 0:26 | 0:36 | 0:33 | 1:02 | 0:15 | 0:55 |
| VarScan | pileup + clean pileup (VarScan, recal) | 0:12 | 0:11 | 0:10 | 0:22 | 0:20 | 0:26 | 0:23 | 0:18 | 0:15 | 0:18 | 0:20 | 0:41 | 0:05 | 0:33 |
| VarScan | varscan default (recal) | 0:07 | 0:06 | 0:05 | 0:12 | 0:11 | 0:14 | 0:12 | 0:07 | 0:07 | 0:09 | 0:10 | 0:20 | 0:03 | 0:15 |
| VarScan | varscan pvalue (recal) | 0:07 | 0:07 | 0:06 | 0:12 | 0:11 | 0:14 | 0:12 | 0:08 | 0:07 | 0:08 | 0:10 | 0:20 | 0:04 | 0:15 |
| VarScan | varscan custom (recal) | 0:07 | 0:06 | 0:05 | 0:12 | 0:10 | 0:14 | 0:12 | 0:07 | 0:07 | 0:08 | 0:10 | 0:19 | 0:02 | 0:15 |
| GATK | GATK Haplotype (recal) | 0:54 | 0:46 | 0:46 | 1:18 | 1:06 | 1:28 | 1:33 | 1:07 | 1:01 | 1:03 | 1:12 | 2:16 | 1:33 | 1:57 |
| GATK | GATK Unified (recal) | 1:51 | 1:45 | 1:47 | 1:51 | 2:08 | 2:02 | 1:58 | 1:44 | 1:55 | 1:55 | 1:47 | 2:22 | 1:39 | 2:09 |
| Both | GATK recal (full pipeline) | 0:22 | 0:21 | 0:18 | 0:44 | 1:31 | 1:41 | 0:43 | 0:34 | 0:26 | 0:37 | 0:33 | 0:59 | 0:14 | 0:55 |
| VarScan | pileup + clean pileup (VarScan, full pipeline) | 0:13 | 0:11 | 0:10 | 0:20 | 0:20 | 0:25 | 0:23 | 0:19 | 0:15 | 0:18 | 0:20 | 0:41 | 0:06 | 0:33 |
| VarScan | varscan default (full pipeline) | 0:07 | 0:06 | 0:06 | 0:12 | 0:11 | 0:14 | 0:12 | 0:07 | 0:08 | 0:08 | 0:10 | 0:21 | 0:03 | 0:17 |
| VarScan | varscan pvalue (full pipeline) | 0:07 | 0:07 | 0:05 | 0:13 | 0:10 | 0:14 | 0:12 | 0:07 | 0:07 | 0:08 | 0:10 | 0:21 | 0:03 | 0:14 |
| VarScan | varscan custom (full pipeline) | 0:07 | 0:06 | 0:06 | 0:11 | 0:11 | 0:14 | 0:12 | 0:08 | 0:06 | 0:08 | 0:10 | 0:20 | 0:03 | 0:15 |
| GATK | GATK Haplotype (full pipeline) | 0:54 | 0:44 | 0:44 | 1:18 | 1:06 | 1:29 | 1:18 | 2:07 | 1:05 | 1:08 | 1:11 | 2:18 | 0:33 | 1:56 |
| GATK | GATK Unified (full pipeline) | 1:47 | 1:52 | 1:41 | 1:57 | 2:02 | 2:05 | 1:58 | 1:50 | 1:54 | 1:54 | 1:45 | 2:22 | 1:43 | 2:11 |

1. Run-times for entire pipeline

| **Pipeline** | **Preprocessing** | **ERR002985** | **ERR002993** | **ERR002994** | **ERR003014** | **ERR003016** | **ERR004078** | **ERR004084** | **SRR013632** | **SRR013635** | **SRR013654** | **SRR013709** | **SRR017908** | **SRR017914** | **SRR018122** |
| --- | --- | --- | --- | --- | --- | --- | --- | --- | --- | --- | --- | --- | --- | --- | --- |
| VarScan: Default | no preprocess | 0:25 | 0:25 | 0:22 | 0:52 | 0:45 | 1:07 | 0:52 | 0:45 | 0:37 | 0:41 | 0:43 | 1:25 | 0:17 | 1:05 |
| VarScan: Default | realign introns | 0:46 | 0:44 | 0:42 | 1:16 | 1:06 | 1:30 | 1:20 | 0:55 | 0:54 | 1:02 | 1:04 | 1:56 | 0:31 | 1:29 |
| VarScan: Default | recalibrate quality scores | 0:46 | 0:46 | 0:51 | 1:34 | 1:15 | 1:48 | 1:36 | 1:12 | 1:05 | 1:19 | 1:17 | 2:27 | 0:32 | 2:08 |
| VarScan: Default | full pipeline | 1:09 | 1:06 | 1:00 | 1:59 | 2:38 | 3:15 | 2:02 | 1:36 | 1:27 | 1:41 | 1:40 | 2:58 | 0:48 | 2:38 |
|  | | | | | | | | | | | | | | | |
| VarScan: pvalue | no preprocess | 0:26 | 0:24 | 0:21 | 0:53 | 0:44 | 1:08 | 0:52 | 0:44 | 0:37 | 0:41 | 0:43 | 1:24 | 0:17 | 1:05 |
| VarScan: pvalue | realign introns | 0:46 | 0:44 | 0:43 | 1:16 | 1:07 | 1:35 | 1:18 | 0:58 | 0:58 | 1:03 | 1:06 | 1:57 | 0:33 | 1:33 |
| VarScan: pvalue | recalibrate quality scores | 0:46 | 0:47 | 0:52 | 1:34 | 1:15 | 1:48 | 1:36 | 1:13 | 1:05 | 1:18 | 1:17 | 2:27 | 0:33 | 2:08 |
| VarScan: pvalue | full pipeline | 1:09 | 1:07 | 0:59 | 2:00 | 2:37 | 3:15 | 2:02 | 1:36 | 1:26 | 1:41 | 1:40 | 2:58 | 0:48 | 2:35 |
|  | | | | | | | | | | | | | | | |
| VarScan: custom | no preprocess | 0:26 | 0:24 | 0:21 | 0:52 | 0:44 | 1:07 | 0:52 | 0:45 | 0:36 | 0:41 | 0:42 | 1:24 | 0:17 | 1:04 |
| VarScan: custom | realign introns | 0:46 | 0:45 | 0:42 | 1:17 | 1:06 | 1:34 | 1:17 | 0:57 | 0:58 | 1:03 | 1:06 | 1:57 | 0:33 | 1:32 |
| VarScan: custom | recalibrate quality scores | 0:46 | 0:46 | 0:51 | 1:34 | 1:14 | 1:48 | 1:36 | 1:12 | 1:05 | 1:18 | 1:17 | 2:26 | 0:31 | 2:08 |
| VarScan: custom | full pipeline | 1:09 | 1:06 | 1:00 | 1:58 | 2:38 | 3:15 | 2:02 | 1:37 | 1:25 | 1:41 | 1:40 | 2:57 | 0:48 | 2:36 |
|  | | | | | | | | | | | | | | | |
| GATK: Unified Genotyper | no preprocess | 1:54 | 1:49 | 1:52 | 3:23 | 2:09 | 3:36 | 2:21 | 2:09 | 2:14 | 3:14 | 2:08 | 2:54 | 1:43 | 2:36 |
| GATK: Unified Genotyper | realign introns | 2:14 | 2:11 | 2:23 | 2:41 | 2:32 | 3:01 | 2:46 | 2:20 | 2:29 | 2:41 | 2:25 | 3:32 | 2:00 | 2:56 |
| GATK: Unified Genotyper | recalibrate quality scores | 2:21 | 2:17 | 2:26 | 2:58 | 2:57 | 3:17 | 3:09 | 2:34 | 2:41 | 2:51 | 2:38 | 3:57 | 2:04 | 3:36 |
| GATK: Unified Genotyper | full pipeline | 2:39 | 2:44 | 2:28 | 3:31 | 4:14 | 4:48 | 3:35 | 3:03 | 3:01 | 3:13 | 2:59 | 4:27 | 2:23 | 4:06 |
|  | | | | | | | | | | | | | | | |
| GATK: Haplotype Caller | no preprocess | 1:04 | 0:56 | 0:52 | 1:44 | 2:22 | 2:04 | 2:51 | 2:25 | 1:28 | 2:11 | 1:48 | 3:40 | 0:51 | 2:24 |
| GATK: Haplotype Caller | realign introns | 1:29 | 1:16 | 1:15 | 2:08 | 1:49 | 2:31 | 2:15 | 1:47 | 1:47 | 2:44 | 2:08 | 4:16 | 1:06 | 2:51 |
| GATK: Haplotype Caller | recalibrate quality scores | 1:24 | 1:18 | 1:25 | 2:25 | 1:55 | 2:43 | 2:44 | 1:57 | 1:47 | 1:59 | 2:03 | 3:51 | 1:58 | 3:24 |
| GATK: Haplotype Caller | full pipeline | 1:46 | 1:36 | 1:31 | 2:52 | 3:18 | 4:12 | 2:55 | 3:20 | 2:12 | 2:27 | 2:25 | 4:23 | 1:13 | 3:51 |

Replicates for same individual highlighted in green and yellow
