# Supplementary material for: Detailed comparison of two popular variant calling packages for exome and targeted exon studies
Source: PeerJ. 2014 Sep 30;2:e600. doi: 10.7717/peerj.600 (PMC4184249; doi:10.7717/peerj.600)
Supplement: Table S13 [file peerj-02-600-s032.doc]

**Table S13: Run Times for 1KG Exome Samples**

1. Run-times for individual steps

| **Pipeline** | **Pre-processing** | **ERR031862** | **ERR031931** | **ERR031956** | **ERR034519** | **ERR034546** | **ERR034553** | **ERR034606** | **SRR098520** | **SRR099528** | **SRR099541** | **SRR100031** | **SRX237141** |
| --- | --- | --- | --- | --- | --- | --- | --- | --- | --- | --- | --- | --- | --- |
| Both | Filter singletons (GATK) | 0:34:00 | 0:50:00 | 0:50:00 | 0:41:00 | 0:39:00 | 0:51:00 | 0:43:00 | 1:01:00 | 0:59:00 | 0:51:00 | 0:30:00 | 0:34:00 |
| Both | sort sample | 0:36:00 | 0:53:00 | 0:53:00 | 0:40:00 | 0:39:00 | 0:44:00 | 0:47:00 | 1:08:00 | 1:05:00 | 0:54:00 | 0:31:00 | 0:32:00 |
| Both | remove duplicates | 0:48:00 | 1:07:00 | 1:13:00 | 12:57:00 | 0:52:00 | 1:01:00 | 1:03:00 | 1:33:00 | 1:21:00 | 1:09:00 | 0:39:00 | 0:42:00 |
| GATK | add read groups + karyotype reorder (GATK) | 0:51:00 | 1:14:00 | 1:23:00 | 1:07:00 | 0:55:00 | 1:11:00 | 1:13:00 | 1:34:00 | 1:24:00 | 1:02:00 | 0:52:00 | 0:44:00 |
| VarScan | pileup + clean pileup (VarScan, no preprocess) | 3:25:00 | 2:56:00 | 6:07:00 | 3:51:00 | 3:27:00 | 4:00:00 | 3:44:00 | 6:25:00 | 3:44:00 | 3:03:00 | 4:51:00 | 3:07:00 |
| VarScan | varscan default (no preprocess) | 1:56:00 | 1:45:00 | 4:41:00 | 2:10:00 | 3:05:00 | 2:35:00 | 2:18:00 | 3:57:00 | 1:59:00 | 1:52:00 | 2:01:00 | 1:49:00 |
| VarScan | varscan pvalue (no preprocess) | 1:58:00 | 1:47:00 | 2:42:00 | 2:15:00 | 2:06:00 | 2:39:00 | 2:20:00 | 2:53:00 | 2:02:00 | 1:52:00 | 2:07:00 | 1:54:00 |
| VarScan | varscan custom (no preprocess) | 1:56:00 | 1:43:00 | 2:35:00 | 2:08:00 | 2:01:00 | 2:23:00 | 2:20:00 | 2:43:00 | 1:55:00 | 2:49:00 | 2:02:00 | 1:48:00 |
| GATK | GATK Haplotype (no preprocess) | 6:59:00 | 16:17:00 | 22:25:00 | 18:39:00 | 11:24:00 | 17:06:00 | 22:52:00 | 28:10:00 | 17:47:00 | 13:30:00 | 16:22:00 | 12:28:00 |
| GATK | GATK Unified (no preprocess) | 4:16:00 | 3:46:00 | 6:18:00 | 4:05:00 | 4:07:00 | 4:33:00 | 4:08:00 | 6:32:00 | 4:06:00 | 3:15:00 | 8:08:00 | 3:26:00 |
| Both | GATK realign | 1:47:00 | 2:15:00 | 3:22:00 | 1:53:00 | 1:46:00 | 2:07:00 | 2:10:00 | 2:46:00 | 2:55:00 | 2:04:00 | 2:34:00 | 1:27:00 |
| VarScan | pileup + clean pileup (VarScan, realign) | 3:25:00 | 2:59:00 | 6:33:00 | 3:31:00 | 4:28:00 | 4:00:00 | 3:47:00 | 6:28:00 | 3:20:00 | 3:01:00 | 3:22:00 | 3:07:00 |
| VarScan | varscan default (realign) | 1:56:00 | 1:45:00 | 2:40:00 | 2:27:00 | 2:06:00 | 2:33:00 | 2:22:00 | 2:58:00 | 1:59:00 | 2:53:00 | 2:04:00 | 1:49:00 |
| VarScan | varscan pvalue (realign) | 1:57:00 | 1:45:00 | 2:45:00 | 2:15:00 | 2:08:00 | 2:36:00 | 2:18:00 | 3:03:00 | 2:02:00 | 1:53:00 | 2:08:00 | 1:53:00 |
| VarScan | varscan custom (realign) | 1:53:00 | 1:42:00 | 2:36:00 | 2:08:00 | 2:03:00 | 2:18:00 | 2:12:00 | 2:48:00 | 1:55:00 | 1:49:00 | 1:58:00 | 1:46:00 |
| GATK | GATK Haplotype (realign) | 7:01:00 | 16:29:00 | 24:12:00 | 18:59:00 | 11:29:00 | 16:43:00 | 23:22:00 | 28:48:00 | 18:46:00 | 13:51:00 | 16:16:00 | 12:15:00 |
| GATK | GATK Unified (realign) | 4:14:00 | 3:38:00 | 6:28:00 | 4:14:00 | 4:05:00 | 4:36:00 | 4:18:00 | 6:50:00 | 3:50:00 | 3:17:00 | 4:09:00 | 3:33:00 |
| Both | GATK recal (w/o realign) | 6:04:00 | 6:40:00 | 10:02:00 | 6:22:00 | 6:10:00 | 7:11:00 | 7:14:00 | 10:29:00 | 8:39:00 | 7:22:00 | 5:15:00 | 5:07:00 |
| VarScan | pileup + clean pileup (VarScan, recal) | 3:37:00 | 3:12:00 | 5:44:00 | 3:59:00 | 3:50:00 | 4:18:00 | 4:22:00 | 7:08:00 | 4:01:00 | 3:41:00 | 3:47:00 | 3:49:00 |
| VarScan | varscan default (recal) | 1:59:00 | 1:45:00 | 2:40:00 | 2:14:00 | 3:08:00 | 2:33:00 | 2:20:00 | 2:58:00 | 2:00:00 | 1:51:00 | 2:02:00 | 1:51:00 |
| VarScan | varscan pvalue (recal) | 1:58:00 | 1:45:00 | 2:45:00 | 2:34:00 | 2:10:00 | 2:36:00 | 2:23:00 | 3:04:00 | 2:03:00 | 1:56:00 | 2:08:00 | 1:54:00 |
| VarScan | varscan custom (recal) | 1:56:00 | 1:42:00 | 2:36:00 | 2:12:00 | 2:07:00 | 2:18:00 | 2:18:00 | 2:48:00 | 1:59:00 | 1:52:00 | 1:57:00 | 1:47:00 |
| GATK | GATK Haplotype (recal) | 16:02:00 | 16:38:00 | 18:48:00 | 17:56:00 | 10:24:00 | 15:07:00 | 22:08:00 | 21:57:00 | 25:33:00 | 11:31:00 | 12:37:00 | 12:16:00 |
| GATK | GATK Unified (recal) | 4:12:00 | 5:31:00 | 6:28:00 | 6:02:00 | 4:13:00 | 4:33:00 | 4:28:00 | 7:01:00 | 3:14:00 | 3:22:00 | 3:56:00 | 3:31:00 |
| Both | GATK recal (full pipeline) | 6:04:00 | 7:03:00 | 9:50:00 | 10:14:00 | 5:31:00 | 7:12:00 | 7:15:00 | 10:19:00 | 7:29:00 | 6:24:00 | 5:09:00 | 6:07:00 |
| VarScan | pileup + clean pileup (VarScan, full pipeline) | 3:37:00 | 3:11:00 | 5:44:00 | 4:23:00 | 3:48:00 | 4:12:00 | 4:06:00 | 7:17:00 | 4:02:00 | 4:41:00 | 3:56:00 | 2:50:00 |
| VarScan | varscan default (full pipeline) | 1:56:00 | 1:46:00 | 2:42:00 | 2:42:00 | 2:06:00 | 2:34:00 | 2:23:00 | 2:59:00 | 2:00:00 | 1:52:00 | 2:07:00 | 1:46:00 |
| VarScan | varscan pvalue (full pipeline) | 1:58:00 | 1:46:00 | 2:44:00 | 4:22:00 | 2:06:00 | 2:38:00 | 2:27:00 | 2:57:00 | 2:04:00 | 1:55:00 | 2:17:00 | 1:52:00 |
| VarScan | varscan custom (full pipeline) | 1:55:00 | 1:42:00 | 2:37:00 | 2:12:00 | 2:06:00 | 2:17:00 | 2:18:00 | 2:47:00 | 1:59:00 | 1:51:00 | 2:03:00 | 2:46:00 |
| GATK | GATK Haplotype (full pipeline) | 16:30:00 | 14:40:00 | 18:55:00 | 18:46:00 | 10:26:00 | 15:34:00 | 21:55:00 | 21:29:00 | 13:59:00 | 11:33:00 | 12:48:00 | 12:23:00 |
| GATK | GATK Unified (full pipeline) | 4:37:00 | 4:00:00 | 6:09:00 | 4:28:00 | 4:07:00 | 4:58:00 | 4:18:00 | 7:18:00 | 3:37:00 | 3:24:00 | 3:57:00 | 3:31:00 |

1. Run-times for entire pipeline

| **Pipeline** | **Pre-processing** | **ERR031862** | **ERR031931** | **ERR031956** | **ERR034519** | **ERR034546** | **ERR034553** | **ERR034606** | **SRR098520** | **SRR099528** | **SRR099541** | **SRR100031** | **SRX237141** |
| --- | --- | --- | --- | --- | --- | --- | --- | --- | --- | --- | --- | --- | --- |
| VarScan: Default | no preprocess | 7:19:00 | 7:31:00 | 13:44:00 | 20:19:00 | 8:42:00 | 9:11:00 | 8:35:00 | 14:04:00 | 9:08:00 | 7:49:00 | 8:32:00 | 6:44:00 |
| VarScan: Default | realign introns | 9:09:00 | 9:56:00 | 15:41:00 | 10:19:00 | 10:33:00 | 11:26:00 | 11:02:00 | 15:55:00 | 11:42:00 | 10:45:00 | 9:53:00 | 8:13:00 |
| VarScan: Default | recalibrate quality scores | 13:38:00 | 14:27:00 | 21:22:00 | 26:53:00 | 15:18:00 | 16:38:00 | 16:29:00 | 24:17:00 | 18:05:00 | 15:48:00 | 12:44:00 | 12:35:00 |
| VarScan: Default | full pipeline | 15:22:00 | 17:05:00 | 24:34:00 | 33:30:00 | 15:21:00 | 18:41:00 | 18:27:00 | 27:03:00 | 19:51:00 | 17:55:00 | 15:26:00 | 13:58:00 |
|  | | | | | | | | | | | | | |
| VarScan: pvalue | no preprocess | 7:21:00 | 7:33:00 | 11:45:00 | 20:24:00 | 7:43:00 | 9:15:00 | 8:37:00 | 13:00:00 | 9:11:00 | 7:49:00 | 8:38:00 | 6:49:00 |
| VarScan: pvalue | realign introns | 9:07:00 | 9:49:00 | 15:36:00 | 21:57:00 | 10:32:00 | 11:19:00 | 10:48:00 | 15:59:00 | 11:42:00 | 9:52:00 | 9:44:00 | 8:15:00 |
| VarScan: pvalue | recalibrate quality scores | 13:37:00 | 14:27:00 | 21:27:00 | 27:13:00 | 14:20:00 | 16:41:00 | 16:32:00 | 24:23:00 | 18:08:00 | 15:53:00 | 12:50:00 | 12:38:00 |
| VarScan: pvalue | full pipeline | 15:24:00 | 17:05:00 | 24:36:00 | 35:10:00 | 15:21:00 | 18:45:00 | 18:31:00 | 27:01:00 | 19:55:00 | 17:58:00 | 15:36:00 | 14:04:00 |
|  | | | | | | | | | | | | | |
| VarScan: custom | no preprocess | 7:19:00 | 7:29:00 | 11:38:00 | 20:17:00 | 7:38:00 | 8:59:00 | 8:37:00 | 12:50:00 | 9:04:00 | 8:46:00 | 8:33:00 | 6:43:00 |
| VarScan: custom | realign introns | 9:03:00 | 9:46:00 | 15:27:00 | 21:50:00 | 10:27:00 | 11:01:00 | 10:42:00 | 15:44:00 | 11:35:00 | 9:48:00 | 9:34:00 | 8:08:00 |
| VarScan: custom | recalibrate quality scores | 13:35:00 | 14:24:00 | 21:18:00 | 26:51:00 | 14:17:00 | 16:23:00 | 16:27:00 | 24:07:00 | 18:04:00 | 15:49:00 | 12:39:00 | 12:31:00 |
| VarScan: custom | full pipeline | 15:21:00 | 17:01:00 | 24:29:00 | 33:00:00 | 15:21:00 | 18:24:00 | 18:22:00 | 26:51:00 | 19:50:00 | 17:54:00 | 15:22:00 | 14:58:00 |
|  | | | | | | | | | | | | | |
| GATK: Unified Genotyper | no preprocess | 7:05:00 | 7:50:00 | 10:37:00 | 19:30:00 | 7:12:00 | 8:20:00 | 7:54:00 | 11:48:00 | 8:55:00 | 7:11:00 | 10:40:00 | 5:58:00 |
| GATK: Unified Genotyper | realign introns | 8:50:00 | 9:57:00 | 14:09:00 | 21:32:00 | 8:56:00 | 10:30:00 | 10:14:00 | 14:52:00 | 11:34:00 | 9:17:00 | 9:15:00 | 7:32:00 |
| GATK: Unified Genotyper | recalibrate quality scores | 13:05:00 | 16:15:00 | 20:49:00 | 27:49:00 | 13:28:00 | 15:31:00 | 15:28:00 | 22:46:00 | 16:42:00 | 14:40:00 | 11:43:00 | 11:10:00 |
| GATK: Unified Genotyper | full pipeline | 15:17:00 | 17:22:00 | 23:40:00 | 32:00:00 | 14:29:00 | 18:04:00 | 17:29:00 | 25:39:00 | 18:50:00 | 15:48:00 | 14:12:00 | 13:37:00 |
|  | | | | | | | | | | | | | |
| GATK: Haplotype Caller | no preprocess | 9:48:00 | 20:21:00 | 26:44:00 | 34:04:00 | 14:29:00 | 20:53:00 | 26:38:00 | 33:26:00 | 22:36:00 | 17:26:00 | 18:54:00 | 15:00:00 |
| GATK: Haplotype Caller | realign introns | 11:37:00 | 22:48:00 | 31:53:00 | 36:17:00 | 16:20:00 | 22:37:00 | 29:18:00 | 36:50:00 | 26:30:00 | 19:51:00 | 21:22:00 | 16:14:00 |
| GATK: Haplotype Caller | recalibrate quality scores | 24:55:00 | 27:22:00 | 33:09:00 | 39:43:00 | 19:39:00 | 26:05:00 | 33:08:00 | 37:42:00 | 39:01:00 | 22:49:00 | 20:24:00 | 19:55:00 |
| GATK: Haplotype Caller | full pipeline | 27:10:00 | 28:02:00 | 36:26:00 | 46:18:00 | 20:48:00 | 28:40:00 | 35:06:00 | 39:50:00 | 29:12:00 | 23:57:00 | 23:03:00 | 22:29:00 |
