# Supplementary material for: Detailed comparison of two popular variant calling packages for exome and targeted exon studies
Source: PeerJ. 2014 Sep 30;2:e600. doi: 10.7717/peerj.600 (PMC4184249; doi:10.7717/peerj.600)
Supplement: Table S15 — Average run-times for are “Full Pipeline” variants (with indel realignment and/or quality score recalibration). VarScan run-time is for VarScan using the default setting. 1KG = selected 1000 genomes project samples. [file peerj-02-600-s034.doc]

**Table S15: Average Run Times for Variant Calling Step for Various Variant Callers**

|  | **1KG Targeted Exon**  **(n=14)** | **1KG Exome**  **(n=12)** | **SRP019719 Exome**  **(n=15)** |
| --- | --- | --- | --- |
| **VarScan**  **(v.2.2.8)** | 0:10 | 2:14 | 1:52 |
| **GATK UnifiedGenotyper**  **(v.2.8.1)** | 1:55 | 4:32 | 4:10 |
| **GATK HaplotypeCaller**  **(v.2.8.1)** | 1:16 | 15:44 | 11:27 |
| **GATK HaplotypeCaller**  **(v.3.1.1)** | 1:33 | 12:49 | 8:51 |
| **Samtools**  **(v.0.1.19)** | 0:26 | 7:04 | 4:51 |
| **Freebayes**  **(v.0.9.14)** | 0:43 |  |  |

Average run-times for are “Full Pipeline” variants (with indel realignment and/or quality score recalibration). VarScan run-time is for VarScan using the default setting. 1KG = selected 1000 genomes project samples.
