# Supplementary material for: Detailed comparison of two popular variant calling packages for exome and targeted exon studies
Source: PeerJ. 2014 Sep 30;2:e600. doi: 10.7717/peerj.600 (PMC4184249; doi:10.7717/peerj.600)
Supplement: Table S16 — Average run-times for are “Full Pipeline” variants (with indel realignment and/or quality score recalibration) for 1000 Genomes Targeted Exon Samples (n = 14). VarScan run-time is for VarScan using the default setting. The GATK HaplotypeCaller was run with the ‘-pairHMM VECTOR_LOGLESS_CACHING’ parameter. Creation of mpileup file not included in calculation above because GATK also has uniquely required pre-processing steps shown in Tables S4–S6; however, we note here that the run-time for creating the mpileup file was 2:24. Separate VarScan variant calls were made using pileup2snp and pileup2indel while joint VarScan variant calls were made using mpileup2snp and mpileup2indel. [file peerj-02-600-s035.doc]

**Table S16: Total Run Times for Variant Calling Step for Various Variant Callers**

1. **1KG Targeted Exon Panel (n=14)**

|  | **Total Run Time** | | |
| --- | --- | --- | --- |
|  | **Separate Variant Calls**  **(Normal .bam)** | **Separate Variant Calls**  **(Reduced Reads .bam)** | **Joint Variant Calls**  **(Reduced Reads .bam)** |
| **VarScan**  **(v.2.2.8)** | 2:22 | NA | 35:56 |
| **GATK UnifiedGenotyper**  **(v.2.8.1)** | 27:01 | NA | 4:06 |
| **GATK HaplotypeCaller**  **(v.3.1.1)** | 21:49 | 20:36 | 11:43 |

1. **1KG Exome (n=12)**

|  | **Total Run Time** | | |
| --- | --- | --- | --- |
|  | **Separate Variant Calls**  **(Normal .bam)** | **Separate Variant Calls**  **(Reduced Reads .bam)** | **Joint Variant Calls**  **(Reduced Reads .bam)** |
| **VarScan**  **(v.2.2.8)** | 26:53 | NA | NA |
| **GATK UnifiedGenotyper**  **(v.2.8.1)** | 54.24 | NA | 11:24 |
| **GATK HaplotypeCaller**  **(v.3.1.1)** | 153:56 | 116:57 | 80:52 |

Average run-times for are “Full Pipeline” variants (with indel realignment and/or quality score recalibration) for 1000 Genomes Targeted Exon Samples (n=14). VarScan run-time is for VarScan using the default setting. The GATK HaplotypeCaller was run with the ‘-pairHMM VECTOR_LOGLESS_CACHING’ parameter. Creation of mpileup file not included in calculation above because GATK also has uniquely required pre-processing steps shown in Tables S4-S6; however, we note here that the run-time for creating the mpileup file was 2:24. Separate VarScan variant calls were made using pileup2snp and pileup2indel while joint VarScan variant calls were made using mpileup2snp and mpileup2indel.
