# Supplementary material for: Detailed comparison of two popular variant calling packages for exome and targeted exon studies
Source: PeerJ. 2014 Sep 30;2:e600. doi: 10.7717/peerj.600 (PMC4184249; doi:10.7717/peerj.600)
Supplement: Table S17 — Average run-times for are “Full Pipeline” variants (with indel realignment and/or quality score recalibration). The GATK (v.3.1.1) HaplotypeCaller was run with the ‘-pairHMM VECTOR_LOGLESS_CACHING’ parameter. 1KG = selected 1000 genomes project samples. [file peerj-02-600-s036.doc]

**Table S17: Impact of running GATK HaplotypeCaller with Reduced Reads**

|  | **Separate Variant Calls**  **(Normal .bam)** | **Separate Variant Calls**  **(Reduced Reads .bam)** |
| --- | --- | --- |
| **1KG Targeted Exon**  **(n=14)** | 1:33 | 1:28 |
| **1KG Exome**  **(n=12)** | 12:49 | 9:44 |

Average run-times for are “Full Pipeline” variants (with indel realignment and/or quality score recalibration). The GATK (v.3.1.1) HaplotypeCaller was run with the ‘-pairHMM VECTOR_LOGLESS_CACHING’ parameter. 1KG = selected 1000 genomes project samples.
