# Supplementary material for: Detailed comparison of two popular variant calling packages for exome and targeted exon studies
Source: PeerJ. 2014 Sep 30;2:e600. doi: 10.7717/peerj.600 (PMC4184249; doi:10.7717/peerj.600)
Supplement: Table S18 — All analyses performed using illumina-100bp-pe-exome-150x dataset [file peerj-02-600-s037.doc]

**Table S18: Links to GCAT Benchmarks**

| **Variant Caller** | **Preprocessing** | **Link** |
| --- | --- | --- |
| **VarScan**  **(v.2.2.8)** | No Preprocessing | http://www.bioplanet.com/gcat/reports/3109-vrqufdpewf |
| **GATK UnifiedGenotyper**  **(v.2.8.1)** | No Preprocessing | http://www.bioplanet.com/gcat/reports/3103-wbhewwrimd |
| **GATK HaplotypeCaller**  **(v.2.8.1)** | No Preprocessing | http://www.bioplanet.com/gcat/reports/3101-pyrhhcvhel |
| **VarScan**  **(v.2.2.8)** | Full Pipeline | http://www.bioplanet.com/gcat/reports/3110-vepctdezwt |
| **GATK UnifiedGenotyper**  **(v.2.8.1)** | Full Pipeline | http://www.bioplanet.com/gcat/reports/3104-pquruqxqje |
| **GATK HaplotypeCaller**  **(v.2.8.1)** | Full Pipeline | http://www.bioplanet.com/gcat/reports/3102-wbnqicnowt |

All analyses performed using illumina-100bp-pe-exome-150x dataset.
