# Supplementary material for: Detailed comparison of two popular variant calling packages for exome and targeted exon studies
Source: PeerJ. 2014 Sep 30;2:e600. doi: 10.7717/peerj.600 (PMC4184249; doi:10.7717/peerj.600)
Supplement: Text S2 [file peerj-02-600-s039.doc]

**Text S2: Commands used for Variant Calling for SRP019719 Samples**

**#variations from 1000 Genomes variant calling are shown in red font**

**#align reads**

/opt/bwa-0.7.5a/bin/bwa aln -t 4 $hg19 $fastq1 > $aln1

/opt/bwa-0.7.5a/bin/bwa aln -t 4 $hg19 $fastq2 > $aln2

/opt/bwa-0.7.5a/bin/bwa sampe $hg19 $aln1 $aln2 $fastq1 $fastq2 > $sam

/opt/samtools-0.1.19/bin/samtools view -bS $sam > $bam

**#remove singletons**

/opt/samtools-0.1.19/bin/samtools view -F 0x04 -b $bam > $filtered_bam

**#sort sample**

/opt/jdk1.6.0_16/bin/java -jar /opt/picard-tools-1.105/SortSam.jar I=$filtered_bam O=$sorted_bam SORT_ORDER=coordinate CREATE_INDEX=True

**#remove duplicates**

/opt/jdk1.6.0_16/bin/java -jar /opt/picard-tools-1.105/MarkDuplicates.jar INPUT=$sorted_bam OUTPUT=$nodup_bam METRICS_FILE=$metrics_file REMOVE_DUPLICATES=true CREATE_INDEX=True

**#create .pileup file (for VarScan)**

/opt/samtools-0.1.19/bin/samtools mpileup -f $hg19 -d 10000 $nodup_bam > $pileup_file

**#VarScan-Default**

/opt/jdk1.6.0_16/bin/java -jar /isi-dcnl/user_data/BIC/Group/Software/Linux/VarScan.v2.2.8.jar pileup2snp $clean_pileup > $varscan_snp

/opt/jdk1.6.0_16/bin/java -jar /isi-dcnl/user_data/BIC/Group/Software/Linux/VarScan.v2.2.8.jar pileup2indel $clean_pileup > $varscan_indel

**#VarScan-Pvalue**

/opt/jdk1.6.0_16/bin/java -jar /isi-dcnl/user_data/BIC/Group/Software/Linux/VarScan.v2.2.8.jar pileup2snp $clean_pileup --p-value 0.05 > $varscan_snp

/opt/jdk1.6.0_16/bin/java –jar /isi-dcnl/user_data/BIC/Group/Software/Linux/VarScan.v2.2.8.jar pileup2indel $clean_pileup --p-value 0.05 > $varscan_indel

**#VarScan-Cons**

/opt/jdk1.6.0_16/bin/java -jar /isi-dcnl/user_data/BIC/Group/Software/Linux/VarScan.v2.2.8.jar pileup2snp $clean_pileup --min-coverage 10 --min-avg-qual 20 --min-reads2 4 --min-var-freq 0.3 > $varscan_snp

/opt/jdk1.6.0_16/bin/java -jar /isi-dcnl/user_data/BIC/Group/Software/Linux/VarScan.v2.2.8.jar pileup2indel $clean_pileup --min-coverage 10 --min-avg-qual 20 --min-reads2 4 --min-var-freq 0.3 > $varscan_indel

**#add read groups (for GATK)**

/opt/jdk1.6.0_16/bin/java -jar /opt/picard-tools-1.105/AddOrReplaceReadGroups.jar INPUT=$nodup_bam OUTPUT=$rg_bam RGLB=1 RGPL=illumina RGPU=barcode RGSM=test CREATE_INDEX=True

**#reorder sample (for GATK)**

/opt/jdk1.6.0_16/bin/java -jar /opt/picard-tools-1.105/ReorderSam.jar I=$rg_bam O=$karyotype_bam REFERENCE=$hg19 CREATE_INDEX=True

**#HaplotypeCaller (v2.8.1 – only for “No Preprocessing” pipeline)**

/opt/jdk1.7.0_45/bin/java -jar /opt/GenomeAnalysisTK-2.8-1-g932cd3a/GenomeAnalysisTK.jar -T HaplotypeCaller -R $hg19 -I $karyotype_bam --genotyping_mode DISCOVERY -stand_emit_conf 10 -stand_call_conf 30 -o $haplotype_vcf --fix_misencoded_quality_scores -fixMisencodedQuals

**#UnifiedGenotyper (only for “No Preprocessing” pipeline)**

/opt/jdk1.7.0_45/bin/java -jar /opt/GenomeAnalysisTK-2.8-1-g932cd3a/GenomeAnalysisTK.jar-T UnifiedGenotyper -R $hg19 -I $karyotype_bam -glm BOTH -stand_emit_conf 10 -stand_call_conf 30 -o $unified_vcf -fixMisencodedQuals

**#HaplotypeCaller (v2.8.1 – “Realign Only”, “Recalibrate Only”, and “Full Pipeline”)**

/opt/jdk1.7.0_45/bin/java -jar /opt/GenomeAnalysisTK-2.8-1-g932cd3a/GenomeAnalysisTK.jar -T HaplotypeCaller -R $hg19 -I $karyotype_bam --genotyping_mode DISCOVERY -stand_emit_conf 10 -stand_call_conf 30 -o $haplotype_vcf

**#HaplotypeCaller (v3.1.1– only used with “Full Pipeline” pre-processing)**

/opt/jdk1.7.0_45/bin/java -jar /opt/GenomeAnalysisTK-3.1-1/GenomeAnalysisTK.jar -T HaplotypeCaller -pairHMM VECTOR_LOGLESS_CACHING -R $hg19 -I $recal_bam --genotyping_mode DISCOVERY -stand_emit_conf 10 -stand_call_conf 30 -o $haplotype_vcf

**#UnifiedGenotyper (“Realign Only”, “Recalibrate Only”, and “Full Pipeline”)**

/opt/jdk1.7.0_45/bin/java -jar /opt/GenomeAnalysisTK-2.8-1-g932cd3a/GenomeAnalysisTK.jar-T UnifiedGenotyper -R $hg19 -I $karyotype_bam -glm BOTH -stand_emit_conf 10 -stand_call_conf 30 -o $unified_vcf

**#indel realignment**

/opt/jdk1.7.0_45/bin/java -jar /opt/GenomeAnalysisTK-2.8-1-g932cd3a/GenomeAnalysisTK.jar -T RealignerTargetCreator -R $hg19 -I $karyotype_bam -o $target_intervals

/opt/jdk1.7.0_45/bin/java -jar /opt/GenomeAnalysisTK-2.8-1-g932cd3a/GenomeAnalysisTK.jar -T IndelRealigner -R $hg19 -I $karyotype_bam -targetIntervals $target_intervals -o $realigned_bam --fix_misencoded_quality_scores -fixMisencodedQuals

**#quality score recalibration**

/opt/jdk1.7.0_45/bin/java -jar /opt/GenomeAnalysisTK-2.8-1-g932cd3a/GenomeAnalysisTK.jar -T BaseRecalibrator -R $hg19 -I $karyotype_bam -knownSites dbsnp_137.hg19.vcf -o $covar_table --fix_misencoded_quality_scores -fixMisencodedQuals

/opt/jdk1.7.0_45/bin/java –jar /opt/GenomeAnalysisTK-2.8-1-g932cd3a/GenomeAnalysisTK.jar -T PrintReads -R $hg19 -I $karyotype_bam -BQSR $covar_table -o $recal_bam -allowPotentiallyMisencodedQuals

**#read reduction**

/opt/jdk1.7.0_45/bin/java -jar /opt/GenomeAnalysisTK-2.8-1-g932cd3a/GenomeAnalysisTK.jar -T ReduceReads -R $hg19 -I $recal_bam -o $reduce_reads_bam
